# Antigen receptor stimulation induces purifying selection against pathogenic mitochondrial tRNA mutations

**DOI:** 10.1172/jci.insight.167656

**Published:** 2023-09-08

**Authors:** Jingdian Zhang, Camilla Koolmeister, Jinming Han, Roberta Filograna, Leo Hanke, Monika Àdori, Daniel J. Sheward, Sina Teifel, Shreekara Gopalakrishna, Qiuya Shao, Yong Liu, Keying Zhu, Robert A. Harris, Gerald McInerney, Ben Murrell, Mike Aoun, Liselotte Bäckdahl, Rikard Holmdahl, Marcin Pekalski, Anna Wedell, Martin Engvall, Anna Wredenberg, Gunilla B. Karlsson Hedestam, Xaquin Castro Dopico, Joanna Rorbach

**Affiliations:** 1 Department of Medical Biochemistry and Biophysics, and; 2 Max Planck Institute Biology of Ageing-Karolinska Institutet Laboratory, Karolinska Institutet, Stockholm, Sweden.; 3 Applied Immunology and Immunotherapy, Department of Clinical Neuroscience, Center for Molecular Medicine, Karolinska University Hospital, Stockholm, Sweden.; 4 Department of Microbiology, Tumor and Cell Biology, Karolinska Institutet, Stockholm, Sweden.; 5 Institute of Infectious Diseases and Molecular Medicine, University of Cape Town, Cape Town, South Africa.; 6 Division of Medical Inflammation Research, Department of Medical Biochemistry and Biophysics, Karolinska Institute, Stockholm, Sweden.; 7 Wellcome Centre for Human Genetics, University of Oxford, Oxford, United Kingdom.; 8 Department of Molecular Medicine and Surgery, Karolinska Institutet, Stockholm, Sweden.; 9 Center for Inherited Metabolic Diseases, Karolinska University Hospital, Stockholm, Sweden.

**Keywords:** Immunology, Metabolism, Adaptive immunity, Mitochondria

## Abstract

Pathogenic mutations in mitochondrial (mt) tRNA genes that compromise oxidative phosphorylation (OXPHOS) exhibit heteroplasmy and cause a range of multisyndromic conditions. Although mitochondrial disease patients are known to suffer from abnormal immune responses, how heteroplasmic mtDNA mutations affect the immune system at the molecular level is largely unknown. Here, in mice carrying pathogenic C5024T in mt-tRNA^Ala^ and in patients with mitochondrial encephalomyopathy, lactic acidosis, stroke-like episodes (MELAS) syndrome carrying A3243G in mt-tRNA^Leu^, we found memory T and B cells to have lower pathogenic mtDNA mutation burdens than their antigen-inexperienced naive counterparts, including after vaccination. Pathogenic burden reduction was less pronounced in myeloid compared with lymphoid lineages, despite C5024T compromising macrophage OXPHOS capacity. Rapid dilution of the C5024T mutation in T and B cell cultures could be induced by antigen receptor–triggered proliferation and was accelerated by metabolic stress conditions. Furthermore, we found C5024T to dysregulate CD8^+^ T cell metabolic remodeling and IFN-γ production after activation. Together, our data illustrate that the generation of memory lymphocytes shapes the mtDNA landscape, wherein pathogenic variants dysregulate the immune response.

## Graphical abstract

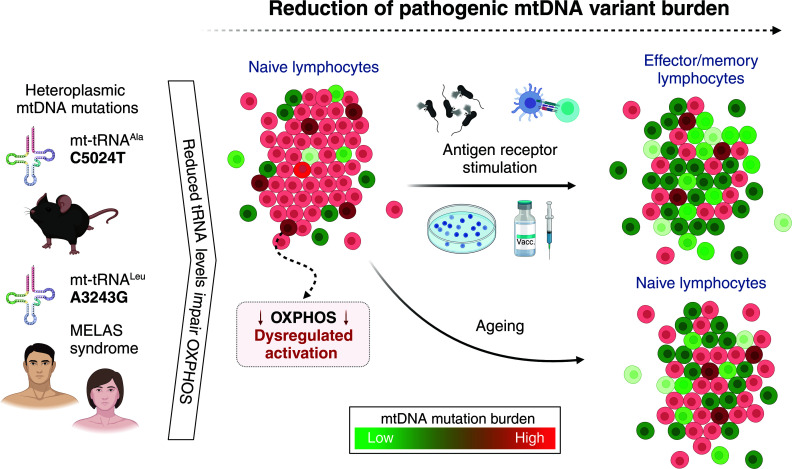

## Introduction

Nuclear and mitochondrial genome (mtDNA) mutations compromising oxidative phosphorylation (OXPHOS) typically affect tissues with a high aerobic demand and can cause multisystemic clinical phenotypes (1, 2). It is well accepted that patients with mitochondrial diseases have an increased infectious disease risk (3, 4), yet cellular and molecular studies of the immune system in such individuals are generally lacking, although this is starting to change (3, 5, 6).

In recent decades, dedicated efforts have been made to explore the interplay between cellular metabolism and immunology, and mitochondria have been found to play critical roles in innate and adaptive lineages (7, 8). OXPHOS is essential for powering lymphocyte activation, proliferation, and differentiation (9–11). Naive T (Tn) lymphocytes require elevated OXPHOS and mitochondrial reactive oxygen species signaling to undergo correct antigen receptor–driven activation (10, 12, 13). Thereafter, T lymphoblasts balance aerobic glycolysis and OXPHOS to fuel rapid proliferation and survival (13), while substantial spare mitochondrial respiratory capacity is essential for antigen-experienced cells to generate long-lived memory cells (9, 14). Recent studies have shown that B cells in germinal centers (GCs) that acquired the highest levels of somatic hypermutation displayed elevated OXPHOS and favored fatty acid oxidation over glycolysis (15, 16). Therefore, mitochondria are key to anamnestic responses. Myeloid cells also depend on mitochondrial metabolism for critical functions, and can be deleteriously affected by mtDNA mutations, impacting innate immunity (17).

Most immunometabolism studies to date have utilized electron transport chain complex inhibitors, or nuclear gene–knockout mouse models that completely abolish OXPHOS function in a targeted subset of immune cells. Unlike these models, maternally inherited pathogenic mtDNA molecules exhibit heteroplasmy within individual cells (18–21). Each cell harbors hundreds to thousands of mtDNA copies. When too many pathogenic molecules are present, affected cells can undergo a metabolic crisis. Accordingly, associated cellular and clinical phenotypes of heteroplasmic mitochondrial mutations may be more heterogeneous than those observed in monogenic mitochondrial diseases (22, 23).

Compared with nuclear genome mutations affecting OXPHOS, which are estimated to be present in 2.9:100,000 individuals, as many as 20:100,000 individuals of European ancestry are predicted to carry pathogenic mtDNA variants (24). Such mtDNA disorders are often debilitating and display highly variable penetrance, pathology, and prognosis. Thus, animal models carrying heteroplasmic mtDNA mutations are useful translational models with the potential to inform molecular studies of immunometabolism. Notably, pathogenic mtDNA mutations have been targeted for elimination with genetic tools in patient cells and to ameliorate disease phenotypes in C5024T (mt-tRNA^Ala^) mice (here studied) (25, 26). Furthermore, 5% differences in C5024T mothers’ heteroplasmy levels were found to be associated with skewed litter heteroplasmy distributions (27), illustrating that above the pathogenic threshold, relatively small changes in heteroplasmy can have important biological effects.

Here, we investigated the effects on immune cells of the C5024T mutation in murine mt-tRNA^Ala^, which impairs OXPHOS and recapitulates different aspects of human mitochondrial diseases, such as cardiomyopathy and altered organ mass ratios in the C57BL/6N background (25, 28, 29). In these animals, mtDNA mutation burden reduction — the percentage by which a tissue had reduced the pathogenic burden compared to baseline (usually a postmitotic tissue with more stable heteroplasmy levels) — is more pronounced in highly proliferative tissues, such as colonic epithelium and blood (27, 29). A deleterious mtDNA molecule harboring a 3.1-kb deletion in a heteroplasmic *Caenorhabditis* *elegans* strain (30, 31) also showed increased selection in the intestine, compared with the body wall muscle. It thus follows that different tissues and cell types and states have variable mutation thresholds, beyond which phenotypes manifest (29). However, the precise triggers for such purifying selection in different tissues remain to be determined.

We sought to extend our observations in the C5024T mouse model to patients with mitochondrial encephalomyopathy, lactic acidosis, stroke-like episodes (MELAS) syndrome who carry different levels of OXPHOS-compromising A3243G in mt-tRNA^Leu(UUR)^. MELAS patients can exhibit severe multisystemic clinical phenotypes when the heteroplasmy burden is above a certain threshold. For example, patients included in this study — with a mutation burden in skeletal muscle greater than 90% — suffered from hypertonia, neurological disorders, stroke-like episodes, cardiomyopathy, and retinitis pigmentosa, among other phenotypes (32–34).

Using C5024T mice and MELAS patient samples, we show that T and B cell populations reduce their pathogenic mtDNA burdens in response to antigen receptor–driven activation and proliferation, illustrating the OXPHOS checkpoints toward immunological memory. We also show that C5024T dysregulates genetic and cellular mechanisms in myeloid and lymphoid cells, supporting the investigation of inherited immunological defects in mitochondrial disease patients.

## Results

### In vivo reduction of C5024T mutation burden by innate and adaptive immune systems.

**Figure 1:**
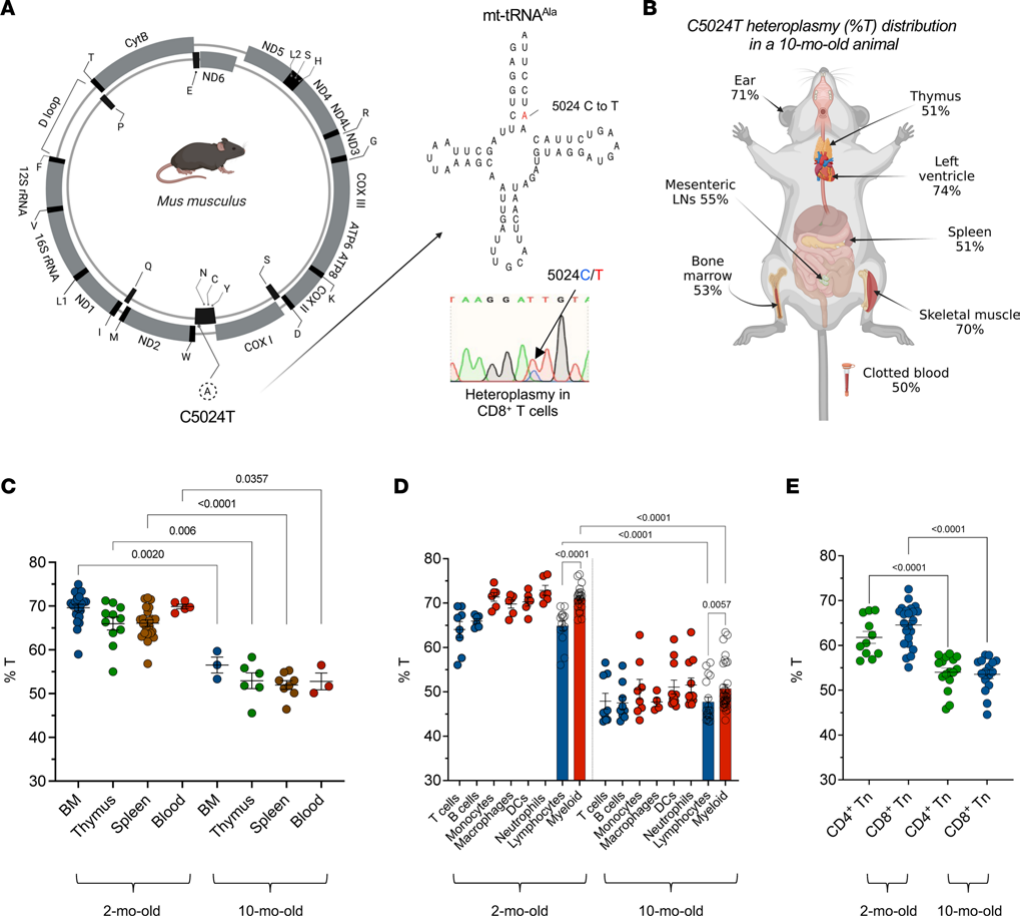
In vivo selection against the C5024T mutation by the innate and adaptive immune systems. (**A**) Schematic representation of murine C5024T in mt-tRNA^Ala^. An electropherogram showing C/T heteroplasmy in purified naive CD8^+^ T cells (inset). (**B**) Pyrosequencing results (%T in total mtDNA) from different anatomical compartments of a representative, 10-month-old C5024T mouse. LN, lymph node. Data representative of *n* = 3 mice. (**C**) Percentage of C5024T heteroplasmy (%T) is shown for selected tissues isolated from 2- and 10-month-old mice (*n* = 3–23 per tissue). (**D**) %T is shown for selected myeloid (red symbols) and lymphoid (blue symbols) lineages FACS isolated from 2- and 10-month-old mice (*n* = 4–9 per cell type). DCs, dendritic cells. Bonferroni’s correction was applied to address the issue of multiple comparisons (α = 0.0125). (**E**) %T is shown for purified naive CD4^+^ and CD8^+^ T cells (*n* = 11–23 per cell type). Cell surface markers and gating strategies used to isolate cell subsets by FACS are detailed in the Methods and supplemental figures. Two-tailed Mann-Whitney tests were used to analyze the data.

C5024T reduces steady-state mt-tRNA levels and causes an OXPHOS deficiency (27, 35), and heteroplasmy levels in subsets of interest were determined using an mtDNA pyrosequencing assay targeting the pathogenic murine C5024T mutation (29) (Figure 1A). C57BL/6N mice with baseline C5024T heteroplasmy (%T) levels of 64%T–80%T (in ear biopsies at weaning) were included in our study. The heteroplasmy burden in ear tissue was not significantly affected by age, being similar between weaning and 16 months of age (29) (Supplemental Figure 1A; supplemental material available online with this article; https://doi.org/10.1172/jci.insight.167656DS1).

In agreement with previous research showing insidious pathology in this model (27, 29), C5024T animals did not manifest any clinical phenotypes up to 10 months of age, compared with wild-type (WT) controls, although 10-month-old male mice had approximately 15% lower bodyweight than WT controls (Supplemental Figure 1B), as previously reported (27, 29). Total white blood cell and erythrocyte counts, and blood Hgb levels, were similar between C5024T and WT animals, as was the cellularity of femur, spleen, and thymus at 2 months of age (Supplemental Figure 1, C and D). Serum IgG titers in 2-month-old animals were also not significantly different (Supplemental Figure 1E).

Compared with tissues with a lower proliferative potential in adulthood — ear, quadriceps, and left heart ventricle — C5024T levels were more greatly reduced in single-cell suspensions from hematopoietic tissues (Figure 1B). Furthermore, we found loss of the variant across hematopoietic tissues to be associated with age, with cells from 10-month-old animals having lower mutation burdens than 2-month-old animals (Figure 1C), recapitulating MELAS patients’ heteroplasmy tissue distribution and decrease with aging (19, 36–40).

In agreement with a recent study of MELAS patients (carrying A3243G in mt-tRNA^Leu^) (41), we observed myeloid lineages from C5024T mice to have higher mutant mtDNA burdens than lymphoid lineages. In C5024T monocytes, macrophages, dendritic cells, and neutrophils isolated by FACS from 2-month-old and 10-month-old animal spleens, myeloid burden reduction was also associated with age (*P* < 0.0001; Figure 1D and Supplemental Figure 2), in agreement with an in silico model that predicted selection against A3243G in hematopoietic stem cells (42). In this respect, age-associated reduction of mutation burdens was also observed in purified naive CD4^+^ and CD8^+^ T cells (Figure 1E).

Together, these data suggest that circulating lymphocyte precursors and stem cells in the thymus and bone marrow undergo selection against the C5024T mutation, in addition to what might be induced peripherally, in agreement with increased oxidative metabolism in proliferating hematopoietic stem cells (43) and bursts of high glucose uptake in common lymphoid progenitors and thymocytes (44).

### Effects of C5024T on bone marrow–derived macrophages.

**Figure 2:**
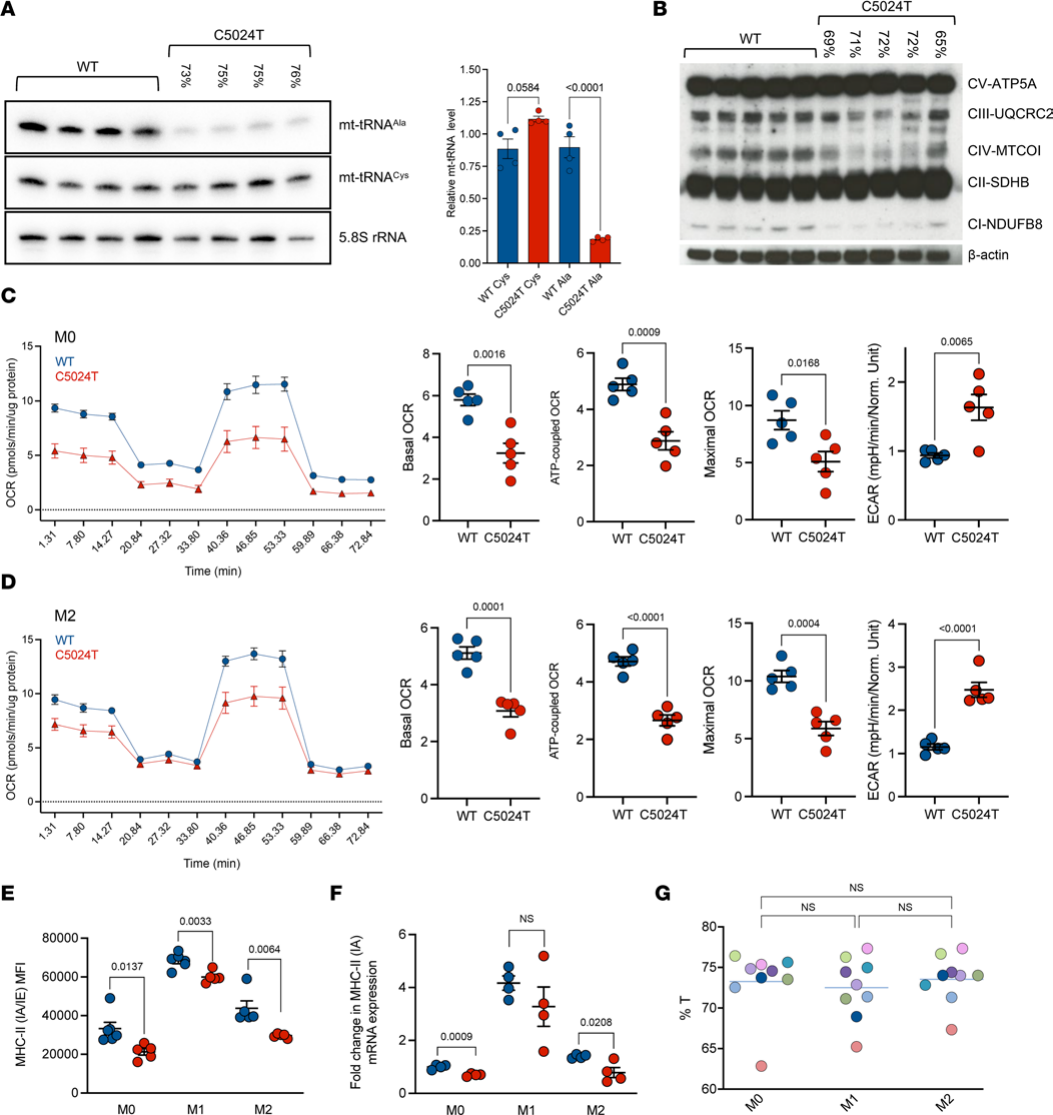
C5024T reduces mt-tRNA^Ala^ levels and compromises OXPHOS in BMDMs. (**A**) Left: Northern blot showing mt-tRNA^Ala^, mt-tRNA^Cys^, and 5.8S RNA levels in C5024T (*n* = 4) and WT (*n* = 4) M0 BMDMs. Ear heteroplasmy levels at weaning are indicated above the respective lanes for mtDNA-mutant animals. Right: Quantification inset. (**B**) Western blot OXPHOS complex profiling in M0 BMDMs from C5024T (*n* = 5) and WT (*n* = 5) animals. Ear heteroplasmy levels at weaning are indicated above the respective lanes for mtDNA-mutant animals. (**C**) Seahorse Mito Stress test of C5024T (*n* = 5) and WT (*n* = 5) M0 BMDMs 24 hours after polarization from M0 macrophages. Data were normalized according to protein amount/well and are representative of 2 independent experiments (Supplemental Table 1). (**D**) Seahorse Mito Stress test of C5024T (*n* = 5) and WT (*n* = 5) M2 BMDMs 24 hours after polarization from M0 macrophages. Data were normalized according to protein amount/well and are representative of 2 independent experiments (Supplemental Table 1). (**E**) Cell surface expression of MHC class II (IA/IE) measured by flow cytometry in BMDMs from C5024T (*n* = 5) and WT (*n* = 5) mice. (**F**) Expression of MHC class II *IA* mRNA in C5024T (*n* = 4) and WT (*n* = 4) BMDM subsets measured by qPCR. Fold-change relative to β-actin. (**G**) Percentage of C5024T heteroplasmy (%T) in M0-, M1-, and M2-polarized cells. No statistically significant changes between the groups or within a mouse were observed in *n* = 9 C5024T samples. Each color represents an individual mouse. Two-tailed, unpaired *t* tests (**A**–**F**) or 2-tailed Mann-Whitney tests (**G**) were used to analyze the data. Bonferroni’s correction was applied to address the issue of multiple comparisons in **G** (α = 0.0166).

As myeloid cells were found to tolerate higher mutation burdens than lymphocytes in vivo, we first investigated whether C5024T affected mitochondrial respiratory capacity in bone marrow–derived macrophages (BMDMs). To do this, we generated M0 (nonpolarized), M1 (proinflammatory), and M2 (alternatively activating) cells from bone marrow progenitors (Supplemental Figure 3, A and B). In agreement with previous studies investigating other tissues in C5024T mice, we observed a reduction in steady-state mt-tRNA^Ala^ levels by Northern blot in M0 BMDMs (Figure 2A). Consequently, we found decreased steady-state levels of OXPHOS complexes I, III, and IV, which contain mitochondria-encoded subunits, in C5024T M0 cells compared with WT controls (Figure 2B).

To determine whether these deficits correlated with reduced OXPHOS capacity at the cellular level, we used the Seahorse Mito Stress test that characterizes the extracellular metabolic flux. We found C5024T M0 and M2 macrophages — which required higher levels of OXPHOS than M1 cells (Supplemental Figure 3C) — to have reduced mitochondrial respiration compared with WT cells, with mutant cultures typified by a high extracellular acidification rate (ECAR) (Figure 2, C and D). Furthermore, we found the expression of MHC class II molecules to be significantly lower at the cell surface in C5024T M0, M1, and M2 cells, compared with WT cells (Figure 2E), although we do not have a mechanistic basis for this observation. These data were mirrored at the mRNA level for class II *IA* but did not reach statistical significance in M1-polarized cells (Figure 2F). Recent work has shown that mitochondrial respiration impacts cell-surface receptor recycling after *Listeria monocytogenes* infection (45), while MHC class II expression has been associated with differences in tumor macrophage metabolism and effector functions (46). Together, these results illustrate that macrophage respiration is compromised by C5024T, which skews these cells toward a glycolytic phenotype that could impact innate immunity.

Importantly, we did not observe significant differences in pathogenic variant burdens in BMDMs polarized to become M0, M1, or M2 cells over an 8-day period (Figure 2G), suggesting that differentiation in the absence of proliferation does not lead to selection at the cellular level in BMDMs, despite the high OXPHOS demands of the M0 and M2 states.

### C5024T heteroplasmy across naive and memory T and B cells.

**Figure 3:**
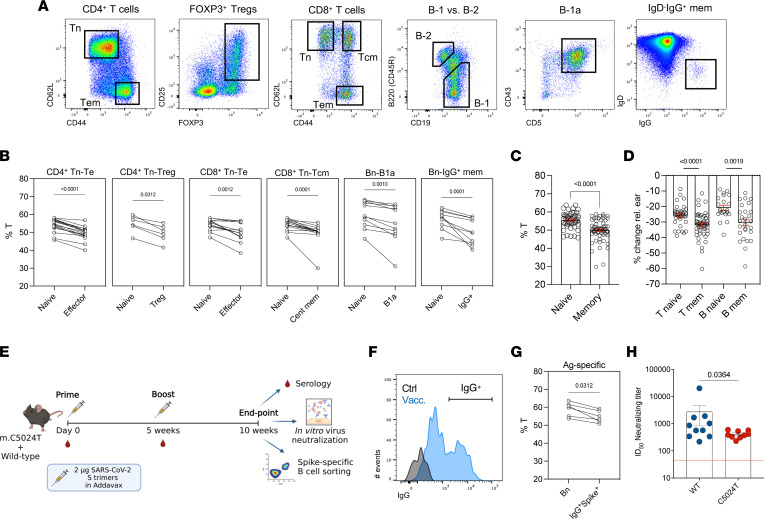
Purifying selection against C5024T in memory T and B lymphocytes in vivo. (**A**) Gates used to FACS isolate naive and memory T and B lymphocyte subsets from C5024T mice (see Methods). All subsets were isolated from the spleen, apart from B-1a cells, which were isolated from the peritoneal cavity. Tn, naive T cells; Tem, effector memory T cells; Tcm, central memory T cells. (**B**) Wilcoxon’s matched-pairs signed-rank test of percentage of C5024T heteroplasmy (%T) for isolated memory lineages compared to their naive counterparts (*n* = 6–17 per cell type). (**C**) %T for all naive and memory lymphocyte subsets in **B** isolated by FACS. (**D**) Percentage change in T (vs. ear) for naive and memory T and B cell lineages in **B**. (**E**) Schematic of the vaccination timeline and readouts. *n* *=* 10 C5024T and *n* = 10 age- and sex-matched WT animals (2 months of age) were immunized. One C5024T mouse was sacrificed during the experiment (week 7) due to development of a wound. (**F**) FACS isolation of vaccine-induced, class-switched S-specific IgG^+^ B cells from the draining lymph nodes of vaccinated C5024T mice, *n* = 6. A representative pseudocolor plot of the antigen-specific (Ag-specific) B cell population from total B-2 cells is shown. Subsets defined using hierarchical gating as naive (CD3^–^CD19^+^B220^+^IgM^hi^IgG^–^S^–^) or Ag-specific memory (CD3^–^CD19^+^B220^+^IgM^–^IgG^+^S^+^). (**G**) Wilcoxon’s matched-pairs signed-rank test of %T in Ag-specific IgG^+^ cells vs. naive cells (Bn). (**H**) ID_50_ neutralizing titers for vaccinated mouse sera. C5024T (*n* = 9) shown in red, WT (*n* = 10) shown in blue. Assay limit of detection ID_50_ = 45. A 1-tailed Mann-Whitney test was used to analyze the data in **H**. Nonparametric 2-tailed Wilcoxon’s matched-pairs signed-rank tests (**B** and **G**) or 2-tailed Mann-Whitney tests (**C** and **D**) were used to analyze the data.

As appropriate OXPHOS function is central to lymphocyte activation, we hypothesized that mutant mtDNA levels in lymphocytes would segregate between naive and memory phenotypes, with antigen-experienced cells having a reduced pathogenic burden. To test this possibility, we isolated naive and memory T and B cell subsets of interest using FACS (Figure 3A and Supplemental Figure 2). As memory cell numbers increase over time, we primarily analyzed the heteroplasmy burden in lymphocytes sorted from 10-month-old animal spleens.

Within the splenic CD4^+^ T cell compartment, we found effector memory (Tem) cells to have a approximately 6% lower mutation burden than their Tn counterparts, having approximately 33% lower C5024T levels than the ear baseline (Figure 3B and Supplemental Figure 4A). Furthermore, FOXP3^+^ regulatory T cells (Tregs) — which have a distinct metabolic profile from that of Tn cells (47, 48) and expressed higher levels of CD44 (Supplemental Figure 4B) — also had reduced mutation burdens compared with naive cells (Figure 3B and Supplemental Figure 4B).

A similar pattern was observed in splenic CD8^+^ T cells, with Tem and central memory (Tcm) subsets having approximately 5% and approximately 6% lower mtDNA mutation burdens, respectively, than naive cells (Figure 3B and Supplemental Figure 4C). By this age, memory CD8^+^ T cells displayed an approximately 30% lower heteroplasmy compared with ear baseline (25, 35).

We next sought to determine whether the same applied to B cells and first isolated fetal-origin B-1a cells from the peritoneal cavity. B-1a cells are known to depend on peripheral self-renewal and have a high capacity for OXPHOS (49). In agreement, we found B-1a cells isolated from the peritoneal cavity to have a higher OXPHOS capacity than did naive B-2 cells from the same site (Supplemental Figure 4D) and had selected more than naive B-2 cells by 2 months of age, which was confirmed in 10-month-old animals (Figure 3B and Supplemental Figure 4, E and F).

Among splenic B cells, class-switched IgG^+^ B-2 cells had a reduced mutation burden compared with unswitched naive cells (Figure 3B and Supplemental Figure 4F). As observed in T cells, several animals showed large heteroplasmy differences between their naive and memory B cells, while in others, differences were more subtle, illustrating how selection pressures vary, perhaps due to environmental effects provoking the immune system.

Across all mice, memory T and B cells subsets had approximately 31% lower %T compared with the ear, while naive cells showed approximately 23% reduction (Figure 3, C and D). Taken together, these results demonstrate that the generation/maintenance of immunological memory in vivo is associated with a reduction in the C5024T variant burden. Importantly, splenic CD4^+^/CD8^+^ T cell ratios, as well as the frequencies of B-2 and NK cells, were similar between naive C5024T mice and WT controls (Supplemental Figure 5A), and we did not observe a significant difference in naive/memory ratios among splenic T cells in 2-month-old animals (Supplemental Figure 5B).

### Inducible mtDNA burden reduction in response to vaccination.

We next sought to determine whether we could induce selection in vivo in response to a specific antigen. To do this, we vaccinated naive, 2-month-old C5024T and WT mice with ancestral SARS-CoV-2 spike (S) glycoprotein trimers (in AddaVax) (50) (Figure 3E). Five weeks after boosting, using a fluorescently labeled trimeric S probe, we FACS-isolated class-switched IgG^+^ S-specific B cells from the draining lymph nodes of a subset (*n* = 6) of vaccinated C5024T mice to determine their mutation burdens (Figure 3F and Supplemental Figure 6A). Compared with S-negative naive B-2 cells, class-switched S-binding cells had a lower mutation burden (Figure 3G and Supplemental Figure 6, B and C), demonstrating the induction of pathogenic burden reduction by vaccination.

With regard to the vaccine response, all but 1 C5024T (*n* = 10) and 1 WT animal (*n* = 9) developed S-specific IgG responses in serum by 5 weeks after the priming dose, and all animals had detectable anti-S IgG 5 weeks after a homologous boost. IgG specific for the neutralization-sensitive epitope, the receptor-binding domain (RBD), was only detectable in serum after the boost dose in all animals and trended toward being reduced in C5024T mice (Supplemental Figure 6, D and E). Despite having similar binding titers at the time points studied, we found that the capacity of vaccinated mouse sera to neutralize pseudotyped virus infection in vitro was lower for C5024T mice (mean ID_50_ 735 vs. 420) 5 weeks after boosting (Figure 3H). This might be apparent at the level of neutralizing activity, but not binding activity, as epitope specificity is less of a determinant for the latter. Indeed, no C5024T mice generated ID_50_ neutralizing titers above a 1:600 serum dilution, while 50% of WT mice did (Figure 3H).

### Proliferation and metabolic stress drive rapid lymphocyte mtDNA variant reduction.

**Figure 4:**
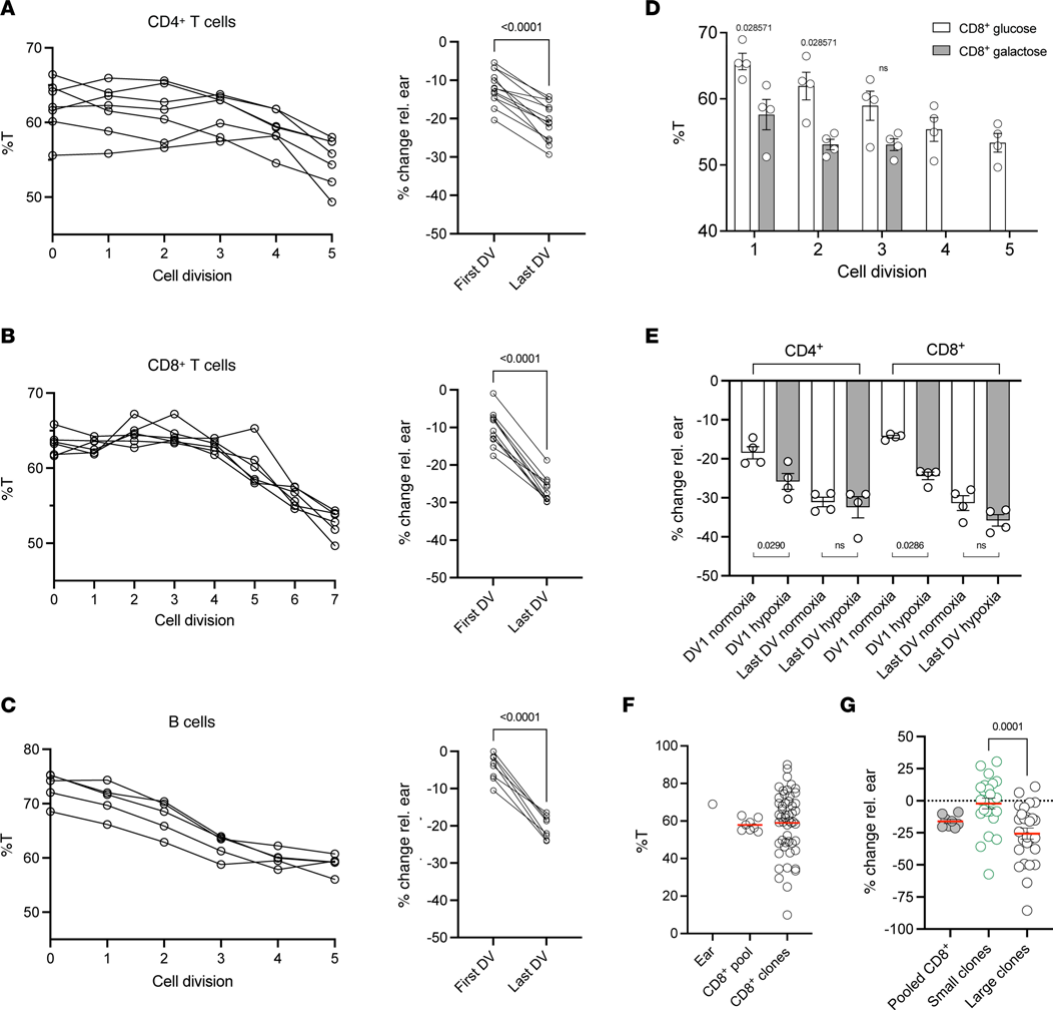
In vitro induction of mtDNA variant selection by antigen-receptor triggering. (**A**) Percentage of C5024T heteroplasmy (%T) is plotted for cells in each division (0–5) sequenced after FACS isolation (*n* = 7). Wilcoxon’s matched-pairs signed-rank test of %T change relative to the ear for undivided CD4^+^ T cells versus cells sorted from the last division after 5 days of stimulation (*n* = 13). (**B**) %T is plotted for cells in each division (0–7) sequenced after FACS isolation (*n* = 7). Wilcoxon’s matched-pairs signed-rank test of %T change relative to the ear for undivided CD8^+^ T cells versus cells sorted from the last division after 5 days of stimulation (*n* = 11). (**C**) %T is plotted for cells in each division (0–7) sequenced after FACS isolation (*n* = 5). Wilcoxon’s matched-pairs signed-rank test of %T change relative to ear for undivided B-2 cells versus cells sorted from the last division after 5 days of stimulation (*n* = 5). (**D**) %T is shown for CD8^+^ T cells TCR stimulated and FACS isolated from different divisions in 5-day galactose (Gal) or glucose (Glu) cultures supplemented with IL-2 (*n* = 4). (**E**) Naive C5024T CD4^+^ and CD8^+^ T cells were activated and cultured under hypoxic (1% O_2_) conditions or normoxia (20% O_2_) for 4 days. Cells from division 1 and cells from the last division were sorted, and the change in %T relative to the ear is displayed. Data from *n* = 4 C5024T samples. (**F**) %T in single CD8^+^ T cell clones from a single adult mouse, compared to the peripheral CD8^+^ pool and the ear baseline. Data representative of multiple (*n* = 5) animals. (**G**) Clonally expanded single CD8^+^ T cells after 7 days of culture after stimulation were classified as belonging to large (>200 cells/well, *n* = 27) or small (<200 cells/well, *n* = 24) clonal populations. Total DNA from each well was then subjected to pyrosequencing. The change in %T relative to ear is plotted for each clonal population and the naive CD8^+^ T cell pool after activation. Nonparametric 2-tailed Wilcoxon’s matched-pairs signed-rank tests (**A**–**C**) or 2-tailed Mann-Whitney tests (**D**, **E**, and **G**) were used to analyze the data.

As the T and B memory pools had selected against C5024T in vivo, we next sought to determine whether we could induce this process in vitro. To this end, we stimulated naive CD4^+^ and CD8^+^ T cells (>64%T) via the T cell receptor (TCR) complex (anti-CD3/anti-CD28) in the presence of IL-2. By FACS-isolating cells from different divisions on day 5, we observed heteroplasmy burden reduction to occur strongly from the third/fourth division onwards (Figure 4, A and B), suggesting that T cells with higher mutation burdens can undergo a few rounds of division under these conditions. Compared with CD4^+^ and CD8^+^ T cells from division 0, cells from the latest division from either lineage had significantly lower C5024T burdens (Figure 4, A and B). This sharp decrease in heteroplasmy between ex vivo naive and the most proliferated CD8^+^ T cells was confirmed by Sanger sequencing (Supplemental Figure 7). Selection against the variant was thus more pronounced in vitro (~17% reduction in stimulated CD8^+^ T cells) than in vivo (~5% difference at 10 months of age).

We also stimulated B-2 cells from the spleens of 2-month-old mice via the B cell receptor (BCR) complex in the presence of anti-CD40 and IL-4. As in T cells, selection was strongly induced in B cells after activation and proliferation (Figure 4C).

To better understand whether a selection plateau/threshold is reached by C5024T T cells stimulated in vitro, we activated cells isolated from mice with lower (<61%T) heteroplasmy burdens in the ear (Supplemental Figure 7B). The data showed that selection in such CD8^+^ T cells reached a plateau of approximately 45%T–50%T under these conditions and approached it more quickly than cells with greater than 64%T — i.e., selection was not observed in subsequent divisions. We note, however, that the threshold (%T) required for different T cell effector functions likely differs between lineages and physiological contexts, while the level of heteroplasmy itself likely affects the number of mitochondrial mechanisms that can be employed, e.g., facilitating differentiation alongside proliferation.

To determine whether metabolic stress impinged on C5024T selection in T cells, we first activated naive CD8^+^ T cells in the presence of galactose (no glucose), which forces cells to rely on OXPHOS to produce ATP. Compared with cells stimulated under normal conditions, CD8^+^ T cells stimulated in galactose cultures underwent fewer divisions in the same time period, while the change in heteroplasmy after the first division was more pronounced (Figure 4D and Supplemental Figure 8A). Similar observations were made in activated naive CD4^+^ T cells (Supplemental Figure 8B), suggesting that a lower mtDNA mutation burden is required for cell division in the absence of glucose.

We also examined whether hypoxic conditions (1% O_2_) affected mtDNA variant selection after activation, as has recently been reported during the in vitro differentiation of primordial germ cell–like cells (51). T cells grown in hypoxic conditions achieved similar proliferation rates to those observed in glucose media, albeit with lower mtDNA variant burdens in earlier divisions compared with WT cells (Figure 4E). As in galactose cultures, heteroplasmy levels in cells from the latest division were comparable between treatment groups, further suggesting a plateau for selection in these conditions.

To further explore the heteroplasmy-proliferation relationship, we analyzed clonal expansion of C5024T CD8^+^ T cells. As reported for other tissues in C5024T mice and mitochondrial disease patients (18, 21, 29, 52, 53), within the peripheral CD8^+^ T cell pool, we found single naive cells to generate clones with a wide range of mutation burdens (Figure 4F). After 7 days of culture after TCR stimulation, we found larger CD8^+^ clonal populations (>200 cells) to have lower C5024T burdens (~25% reduction vs. ear) than smaller ones (<200 cells) (~2% reduction) (Figure 4G). These results indicate that even high-mutation burden cells — e.g., greater than 75%T — can proliferate, although they do so with slower kinetics, and thus may be outcompeted in vivo. Indeed, compared with WT cells, a greater proportion of C5024T CD4^+^ and CD8^+^ T cells 5 days after activation were in the latest division (Supplemental Figure 8C), supporting the idea that cells with higher mutation burdens remain in early divisions or die.

Together, our results suggest that proliferation of cells with low pathogenic heteroplasmy burdens is a mediator of variant loss in the lymphocyte pool. However, we do not exclude additional mechanisms, such as asymmetric division, differences in mtDNA replication, and mitophagy, that may play a role in variant loss.

### Dysregulated metabolic remodeling during C5024T T cell activation.

**Figure 5:**
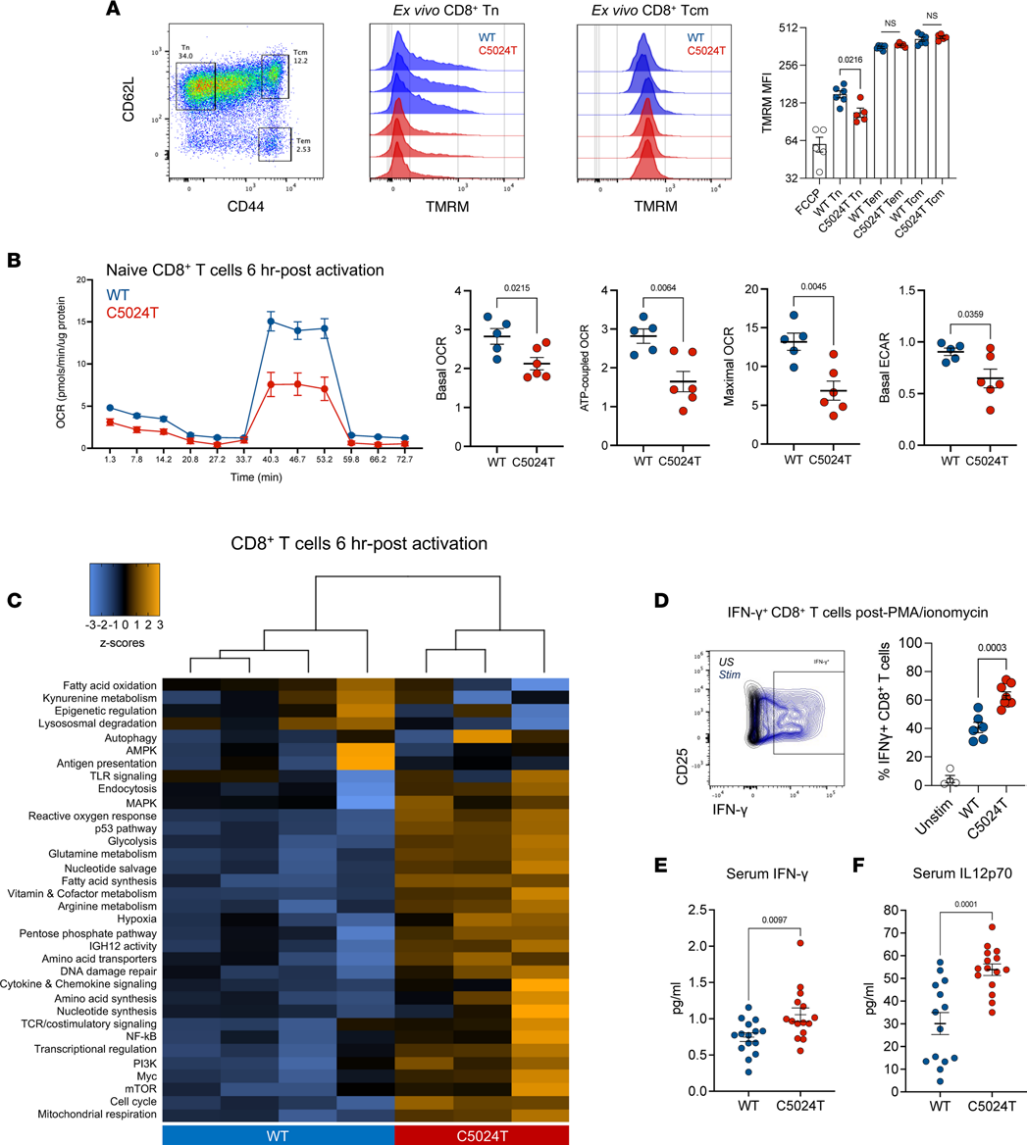
C5024T dysregulates CD8^+^ T cell metabolic remodeling after activation. (**A**) Mitochondrial membrane potential was measured using TMRM in ex vivo CD8^+^ T cell subsets from the spleens of C5024T (*n* = 5) and WT (*n* = 6) mice. Representative data from *n* = 3 per genotype are shown. (**B**) Seahorse Mito Stress test of ex vivo naive CD8^+^ T cells isolated from adult mice (*n* = 6 C5024T*, n* = 5 WT) and stimulated for 6 hours with anti-CD3/anti-CD28 beads and IL-2 before analysis. Data representative of 2 independent experiments (Supplemental Table 1). (**C**) nanoString metabolic gene module analysis of mRNA isolated from CD8^+^ T cells from young C5024T (*n* = 3) and WT (*n* = 4) mice stimulated for 6 hours with anti-CD3/anti-CD28 and IL-2. Hierarchical clustering of principle component differences in normalized gene expression per module is shown. One C5024T sample (from *n* = 4) did not pass QC and was excluded. (**D**) Proportion of IFN-γ^+^ cells in the latest division, assessed by flow cytometry after PMA/ionomycin restimulation of CD8^+^ T cells previously expanded for 5 days with anti-CD3/anti-CD28 and IL-2. C5024T (*n* = 8) and WT (*n* = 6). (**E**) Serum IFN-γ levels in C5024T (*n* = 15) and WT (*n* = 15) animals measured by electrochemiluminescence. (**F**) Serum IL-12p70 levels in C5024T (*n* = 14) and WT (*n* = 15) animals similarly measured. Two-tailed, unpaired *t* tests (**A**) or 2-tailed Mann-Whitney tests (**B** and **D**–**F**) were used to analyze the data.

We next sought to determine whether purifying selection in T cells correlated with an improvement in their respiratory capacity. To do this, we stained ex vivo splenocytes with the mitochondrial membrane potential (MMP) marker, tetramethylrhodamine methyl ester perchlorate (TMRM), for analysis by flow cytometry. We found naive CD8^+^ T cells from C5024T mice to have lower MMP than WT cells, although this difference was abrogated in memory cells (Figure 5A), supporting the notion that a reduction in C5024T burden helps restore appropriate MMP. The respiratory deficit of naive CD8^+^ T cells was confirmed using the Seahorse Mito Stress test. In unstimulated conditions ex vivo, naive CD8^+^ T cells from C5024T mice showed reduced basal and maximal oxygen consumption rate (OCR) compared with WT cells, although differences were subtle, perhaps because of the low metabolic demands of unactivated T cells (Supplemental Figure 8D). However, after 6 hours of anti-CD3/anti-CD28 stimulation, C5024T cells had reduced basal and maximal OCR, ATP-coupled OCR, and ECAR, compared with WT CD8^+^ T cells (Figure 5B), illustrating that the OXPHOS burst of T cell activation is particularly impaired by the mutation.

To further determine how naive CD8^+^ T cell cultures were metabolically dysregulated by C5024T, mutant and WT cells were stimulated via the TCR for 0, 6, and 96 hours before metabolic gene expression analysis using nanoString (54). Within 6 hours of stimulation, several genes and processes important for CD8^+^ T cell effector functions were strongly upregulated, compared with in unstimulated (0 hour) cells (Supplemental Figure 9A). Gene module analysis revealed the overall metabolic remodeling of early T cell activation (Supplemental Figure 9, B and C), which is largely consistent with previous research (8, 55). With regard to genotype, in contrast to the 0- and 96-hour time points, after 6 hours of activation, C5024T and WT CD8^+^ T cells clustered separately (Figure 5C and Supplemental Figure 9B), further supporting the idea that the early activation response is impaired by C5024T, and that genotypic differences are less apparent in unactivated (0 hour) cells and after 4 days of proliferation-associated burden reduction.

After 6 hours, C5024T cells had an upregulated metabolic and inflammatory profile compared with WT cells, with increased expression of genes involved in mitochondrial respiration, the cell cycle, cytokine and chemokine signaling, and the ROS and hypoxia responses (Supplemental Figure 9D). In contrast, genes involved in epigenetic remodeling were less expressed in C5024T than in WT cells. These results illustrate dysregulated metabolic reprogramming of naive C5024T CD8^+^ T cells after TCR activation. The upregulation of nuclear-encoded metabolic genes observed in C5024T cells likely occurs as a compensatory mechanism to improve mitochondrial function in T cells with more severely impaired OXPHOS, as suggested by similar results from fibroblasts from MELAS (A3243G) patients (56), and is consistent with recent research illustrating a hypermetabolic state of OXPHOS-deficient cells (57).

Given these results, to determine whether functional deficits persisted in the CD8^+^ population after burden reduction, we restimulated 5-day-activated cultures with PMA/ionomycin. Six hours after restimulation, we found a greater proportion of C5024T CD8^+^ T cells from the latest division to produce IFN-γ, compared with those from WT animals (Figure 5D), supporting the notion that C5024T drives a proinflammatory T cell phenotype. Increased IFN-γ production has previously been associated with mitochondrial insufficiency and may represent an OXPHOS-glycolysis imbalance (13, 58). In light of this, to determine whether C5024T influenced systemic immunophenotypes, we analyzed cytokine levels in the serum using multiplex electrochemiluminescence. We found serum IFN-γ and particularly IL-12p70 levels to be elevated in naive mutant animals, compared with WT controls (Figure 5, E and F). IL-10 levels in the serum were also marginally increased (*P* = 0.04, data not shown) in C5024T mice. The levels of IL-1β, IL-2, IL-4, IL-5, IL-6, CXCL1, and TNF-α were not statistically different between genotypes (Supplemental Data Values).

### Reduction of pathogenic A3243G mtDNA burden in memory T and B cells from human MELAS patients.

**Figure 6:**
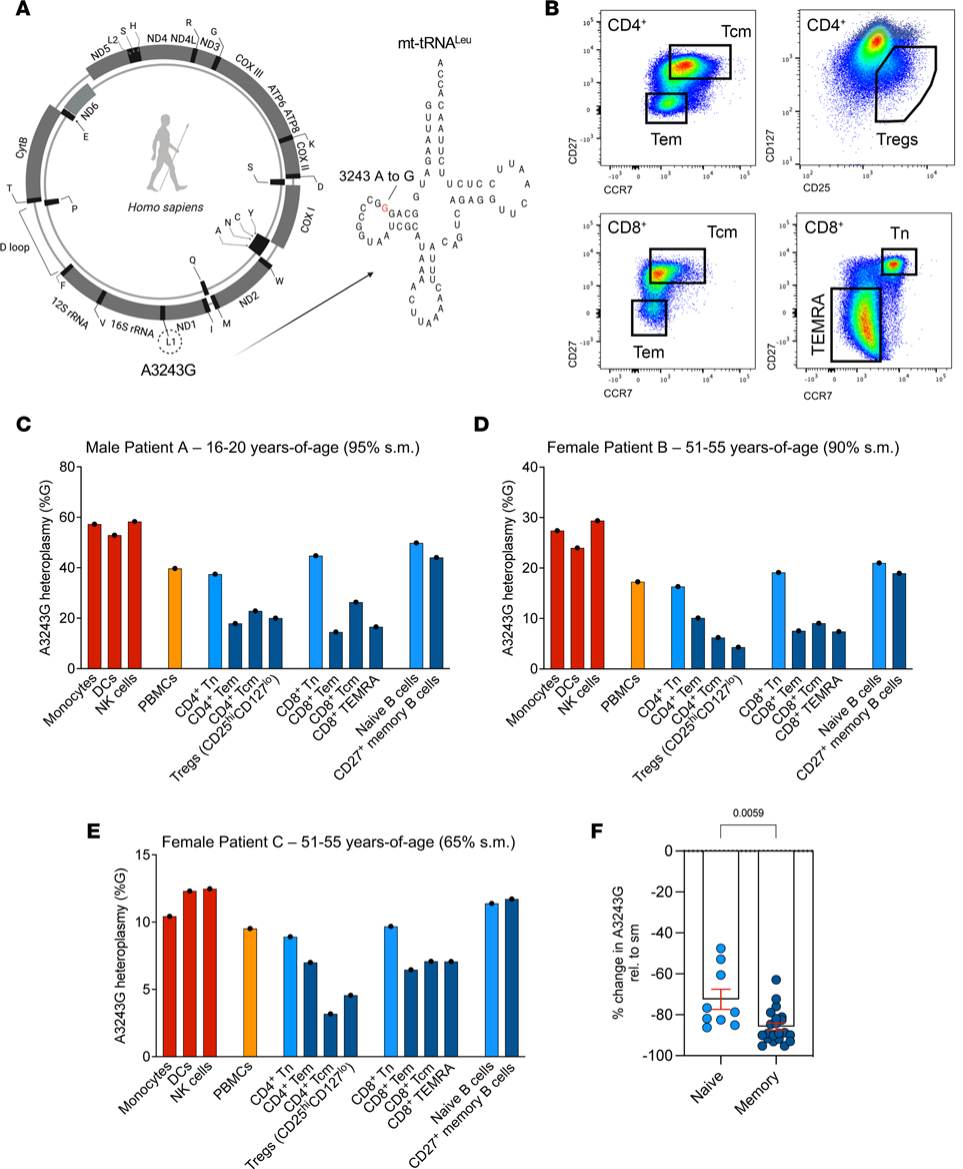
Reduction of A3243G burden in memory T and B cells from human MELAS patients. (**A**) Schematic representation of human A3243G in mt-tRNA^Leu^. (**B**) FACS strategy for isolation of key human T lymphocyte populations from MELAS patients. Key T cell subsets are shown; the sorting strategy is shown in the supplemental figures. (**C**) Percentage change in A3243G heteroplasmy in immune cells, relative to skeletal muscle (s.m.) biopsy is shown for Patient A (male, 16–20 years of age). Skeletal muscle heteroplasmy level in patient: 95%. (**D**) As in **C** for Patient B (female, 51–55 years of age). Skeletal muscle heteroplasmy level in patient: 90%. (**E**) As in **D** for Patient C (female, 51–55 years of age). Skeletal muscle heteroplasmy level in patient: 65%. (**F**) Percentage change in heteroplasmy (relative to s.m.) for all naive and memory T and B subsets isolated from Patients A, B, and C. Temra, terminally differentiated effector memory cells re-expressing RA. Two-tailed Mann-Whitney tests were used to analyze the data.

Given our observations in the mouse model, we sought to determine whether human memory T and B cells carrying a different pathogenic heteroplasmic mtDNA variant — also in an mt-tRNA gene — exhibited the same purifying selection. For this purpose, we used FACS to examine different peripheral blood immune cell types from 3 unrelated MELAS patients carrying the A3243G mutation in mt-tRNA^Leu^ (Figure 6, A and B, and Supplemental Figures 10 and 11). Patients exhibited a range of clinical characteristics and heteroplasmy levels, as detailed in Table 1.

In MELAS Patient A (male, 16–20 years of age), who had a whole-blood A3243G mutation burden of 50% (compared with 95% burden in skeletal muscle), we found isolated myeloid lineages to have higher mutant mtDNA burdens than did T and B cells (Figure 6C). In this patient, freshly isolated monocytes, dendritic cells, and NK cells had approximately 40% lower A3243G burden compared with skeletal muscle, while total PBMCs showed approximately 50% lower levels.

Within T cell subsets from Patient A, both naive CD4^+^ and CD8^+^ cells had a higher mutation burden (~60% reduction) than their respective Tem, Tcm, and terminally differentiated Tem cells re-expressing RA (Temra) subsets (each approaching an ~80% reduction) (Figure 6C). Here, CD8^+^ Tem cells showed an approximately 30% greater burden reduction than their Tn counterparts (Figure 6C). Within the B cells, we also observed naive B cells to have a higher mutation burden (-48%) than CD27^+^ memory B cells, with the latter showing a 63% reduction.

In unrelated Patient B (female, 51–55 years of age with 90% heteroplasmy burden in skeletal muscle, 25% in blood), the same patterns were observed. However, as Patient B was considerably older than Patient A, burden reduction was more pronounced (Figure 6D). Monocytes, dendritic cells, and NK cells from Patient B showed an approximately 70% burden reduction (relative to skeletal muscle) at sampling (compared with 40% in Patient A), while PBMCs showed an approximately 80% reduction.

Despite the strong selection against A3243G across the hematopoietic system in this patient, naive T and B cells still had a clearly higher pathogenic variant burden than their memory counterparts. For example, Tregs in this patient showed a 95% reduction to baseline, while naive CD4^+^ T cells reduced their burden by 81%. It is noteworthy that knockout of mitochondrial complex III abolishes Treg-mediated suppression (59). CD27^+^ memory B cells also displayed greater burden reduction than did naive B cells (84% vs. 76% reduction) (Figure 6D).

In unrelated Patient C (female, 51–55 years of age), a similar pattern was observed (Figure 6E). However, this patient had a much lower baseline level of mutation (65% in skeletal muscle) compared with the other patients and differences were more subtle, in line with a less severe clinical history (Table 1). The level of mutation in total PBMCs in this patient was 9%, indicating this individual had already undergone strong selection against the variant across the hematopoietic system by the time of sampling. Despite this, as in the other 2 patients, myeloid lineages selected against the variant less than did total PBMCs, while CD4^+^ and CD8^+^ memory T cell subsets again had a reduced level of mutation compared with naive cells. CD4^+^ Tcm cells in this patient had a heteroplasmy level of just 3%, illustrating continual burden loss in T cell lineages. We did not, however, observe a difference between naive and memory B cells in Patient C, perhaps because an approximately 10% mutation burden in B cells approaches levels at which the variant is not pathogenic, in agreement with B cells selecting against the variant less strongly than T cells in the patients with higher mutation burdens. Thus, across all 3 MELAS patients, memory lymphocyte subsets had selected against the pathogenic variant more strongly than their naive counterparts (Figure 6F). The same was observed for lymphoid compared with myeloid lineages (Supplemental Figure 11C), as previously reported (41).

Therefore, as for C5024T, the human A3243G mutation in mt-tRNA^Leu^ is reduced in frequency in memory lymphocytes, compared with naive cells, illustrating how OXPHOS capacity is a fundamental determinant of lymphocyte activation and maintenance, supporting the presence of inherited immune dysregulation in MELAS patients.

## Discussion

Together, our results demonstrate the high metabolic demands of adaptive immunity, which purges the pathogenic mtDNA variants as OXPHOS insufficiency takes its toll on lymphocyte development and maintenance. However, the (i) specific triggers or checkpoints of such burden reduction, (ii) the relative contributions of these (e.g., central tolerance signaling vs. peripheral antigen encounter), and (iii) whether these mutations impact T and B cell responses, had not previously been studied.

Data from ex vivo mouse and human immune cell subsets revealed that the lymphocyte naive-memory transition was accompanied by a reduction in pathogenic C5024T and A3243G variant burdens in vivo. Therefore, after a lymphocyte has undergone V(D)J recombination and enters circulation, further genetic selection, this time in mtDNA, occurs during the generation/maintenance of immunological memory (and presumably in tandem with somatic hypermutation in B cells), as supported by recent research (60). Certain T and B cells, like hematopoietic stem cells, are predicted to undergo self-renewal for the lifetime of the organism (61) and require metabolic remodeling to achieve optimal function and maintain clonal longevity. Indeed, antigen-experienced B cells are known to require elevated OXPHOS to achieve high-affinity antibody maturation in the GC (15), which may contribute to the reduced neutralizing titers we observed in C5024T mice in response to SARS-CoV-2 S vaccination. Thus, given our observations, the study of vaccine-induced responses, their efficacy and duration, especially taking age into consideration, seems particularly relevant for MELAS and other mitochondrial disorders.

Pathogenic burden reduction is predicted to progress until a tolerable level is achieved by a cell lineage and state. Our data show that the pathogenic mtDNA variants of C5024T mice and MELAS patients remain under selective pressure well into adulthood in lymphocytes, especially if the mutation burden is high (e.g., >75% mutation) to begin with. Strikingly, MELAS Patient C had just 3% of the pathogenic variant in CD4^+^ Tem cells at age 51–55, with 65% in skeletal muscle. In humans, the peripheral naive/memory T cell ratio is approximately equal by the age of 30 and thymic involution makes peripheral self-renewal more important for naive and memory T lymphocyte maintenance in adulthood and old age (62). Therefore, peripheral self-renewal of ever fewer clonal T and B lineages is predicted to maintain immunity in MELAS patients, as cells with higher mutation burdens fail to fully differentiate. Accordingly, the study of the adaptive immune system in mitochondrial disease patients with systems biology approaches is warranted. Moreover, as recent work suggests that a hypermetabolic state due to OXPHOS deficiency shortens cell longevity, it will be interesting to monitor immune response duration in C5024T mice and human patients (57), as well as how differences in mtDNA copy number between immune lineages and differentiation/effector states impinge on heteroplasmy.

With regard to immunological phenotypes associated with the C5024T mutation, our transcriptional and cellular data together indicate that the mutation dysregulates macrophage and T and B cell responses at transcriptional, protein, and cellular levels, e.g., by dampening epigenetic regulation of gene expression (55) and increasing IFN-γ production by activated CD8^+^ T cells (13, 58). Elevated IL-12p70 and IFN-γ levels observed in sera from C5024T mice illustrate systemic immunophenotypes associated with mtDNA mutations, which require further study in mitochondrial disease patients. Furthermore, as myeloid cells had a higher tolerance for the C5024T and A3243G mutations, it will also be important to decipher how deficits in innate cells contribute to any differences in disease susceptibility in these mice and MELAS patients.

We show that burden reduction is readily inducible in vitro by triggering lymphocyte proliferation by TCR or BCR ligation, which facilitates the study of mtDNA selection mechanisms in single primary cells. Indeed, as burden reduction was observed in vaccine-induced B cells approximately 5 weeks after immunization, variant loss takes place shortly after antigen encounter, although homeostatic proliferation and tonic signaling in the periphery may contribute to ongoing burden reduction in newly formed memory cells over longer periods, especially in humans. Our results suggest that proliferation of cells with low burdens is a major mechanism driving variant loss at the population level, as C5024T CD8^+^ T cell clones with high burdens proliferated more slowly than did clones with low burdens (Figure 4G). Nevertheless, further studies are needed to determine whether mutant mtDNA can be targeted for removal during different phases of a cell’s life and context, and how mtDNA segregates during cell division. Indeed, a recent study reported that low oxygen tension contributes to tissue-specific mutation load segregation (51), and we noticed upregulated hypoxia pathway genes in activated C5024T CD8^+^ T cells. By activating T cells in galactose media and hypoxic conditions, we showed that the metabolic environment can alter the threshold for lymphocyte division. Thus, it is likely that the extent of burden reduction is influenced by the metabolic and inflammatory conditions in which activated immune cells work. It has also recently been reported that metabolite restriction can affect mtDNA replication (63), an area that merits further exploration in lymphocytes.

Together, our data reveal a traceable immunophenotype of human mitochondrial disease and demonstrate the deregulatory effect mt-tRNA mutations can have on innate and adaptive immune cells. Thus, a better understanding of immunity in mitochondrial disease patients may inform not only upon clinical phenotypes, but also how the immune system evolves when a proportion of the pool has reduced mitochondrial respiratory capacity. Our study supports the notion that C5024T mice are a translationally valuable model that could be used alongside patient samples to investigate energetic bottlenecks in hematopoiesis and how these impact health and disease.

## Methods

### Animal breeding and housing.

C57BL/6NCrl mice harboring a point mutation (C5024T) in mt-tRNA^Ala^ were generated as previously described (27). As C5024T is maternally transmitted, all littermates have different mutation burdens. WT controls with the same nuclear background were used in an age- and sex-matched manner, and animals were housed in a 12-hour light/dark cycle with ad libitum water and standard chow. Male mice were used throughout for in vivo studies, as they were previously reported to have a lower body weight than WT mice (http://www.informatics.jax.org/allele/MGI:5902095), whereas females were unaffected. For in vitro experiments, male and female mice were used. Mice were sacrificed using CO_2_ and cervical dislocation at experimental endpoints. Animals with an ear biopsy heteroplasmy level between 64% and 80% at weaning were included in this study, unless otherwise indicated for specific experiments. Mouse blood parameters were measured using a KX-21N hematology analyzer (Sysmex). All animal procedures were carried out in accordance with the 3Rs principles.

### Human samples.

MELAS patients under the care of the Center for Congenital Metabolic Diseases at Karolinska University Hospital were invited to participate in this study by their attending clinicians. On the day of clinic visit, peripheral blood samples were drawn into lithium heparin–coated tubes and processed for PBMC isolation within 1 hour of venesection. An aliquot of plasma was removed prior to density gradient centrifugation and stored at –80°C. PBMCs were isolated using Lymphoprep (StemCell Technologies) and washed with RPMI-1640 (Cytiva HyClone) plus 10% FBS before cryopreservation at –80°C in FBS plus 10% DMSO (Sigma-Aldrich).

### Murine immunophenotyping.

Primary tissues of interest were dissected from mice ex vivo. To obtain single-cell suspensions, tissues were disrupted and intact femurs flushed in Dulbecco’s PBS (D-PBS) with 0.2% FBS and filtered through a 70 μm filter before counting with trypan blue using a Countess cell counter (Invitrogen). Body cavity washing was achieved by instilling 8 mL cold D-PBS with 0.2% FBS into the peritoneum before aspiration. For indicated experiments, total B and Tn cell subsets were purified from splenic single-cell suspensions using magnetic negative selection: EasySep Mouse B cell isolation kit (StemCell Technologies) and CD4^+^ or CD8a^+^ T cell isolation kits (Miltenyi Biotec). Otherwise, single-cell suspensions were resuspended to the appropriate concentration and stained with fluorescently labeled monoclonal antibodies in the dark for 45 minutes at 4°C. For intracellular protein staining, samples were permeabilized, fixed, and stained using the Foxp3/Transcription Factor Staining Buffer Set (Invitrogen) according to the manufacturer’s instructions. Aqua or near-IR live/dead dyes (Invitrogen) were used to discriminate viable cells, while CFSE (eBioscience) or Cell Trace Violet (Thermo Fisher Scientific) dye was used to assess proliferation. Cells were fixed for analysis (but not FACS) using 4% paraformaldehyde. Live-discriminated murine cells were defined as follows: Monocytes: CD3^–^CD19^–^CD11b^+^Ly6G^–^Ly6C^hi^. Neutrophils: CD3^–^CD19^–^CD11b^+^Ly6G^+^. Dendritic cells: CD3^–^CD19^–^CD11b^+^CD11c^+^MHC-II^+^. NK cells: CD3^–^CD19^–^NK1.1^+^. CD4^+^ Tn: CD3^+^CD4^+^CD44^–^CD62L^+^. CD4^+^ Tem: CD3^+^CD4^+^CD44^hi^CD62L^–^. CD4^+^ Tregs: CD3^+^CD4^+^CD44^+^CD25^+^FOXP3^+^. CD8^+^ Tn: CD3^+^CD8^+^CD44^–^CD62L^+^. CD8^+^ Tem: CD3^+^CD8^+^CD44^hi^CD62L^–^. CD8^+^ Tcm: CD3^+^CD8^+^CD44^hi^CD62L^+^. γδ T cells: CD19^–^CD3^+^γδ-TCR^+^. B-1a: CD19^hi^B220^lo^CD5^+^CD43^+^IgM^+^. Naive B-2: CD3^–^CD19^+^B220^+^IgD^hi^IgM^hi^IgG^–^. Memory B-2: CD3^–^CD19^+^B220^+^IgD^–^IgM^–^IgG^+^. S-specific B-2: CD3^–^CD19^+^B220^+^IgM^–^IgG^+^S^+^. Antibodies are described in Supplemental Table 1. Prefusion-stabilized (ancestral) SARS-CoV-2 S trimers and the RBD were produced as in Hanke et al. (64). The S probe was used at a 1:1000 dilution prior to staining for other cell-surface antigens previously titrated.

### MELAS immunophenotyping.

After thawing frozen PBMC aliquots, cells were washed with RPMI plus 10% FBS before resting at 37°C (5% CO_2_) for 30 minutes. Cells were prestained for CCR7 during incubation, as recommended for this antigen; all additional staining was carried out at 4°C after PBMCs were allowed to rest after thawing. Cells were stained with Live/Dead dye for 15 minutes prior to surface staining.

The following markers were used to classify human cells: Monocytes: CD45^+^CD3^–^CD20^–^CD56^–^CD14^+^CD16^+^HLA-DR^+^. Dendritic cells: CD45^+^CD3^–^CD20^–^CD56^–^CD14^–^CD16^+^HLA-DR^+^. NK cells: CD45^+^CD3^–^CD20^–^CD56^+^. CD4^+^ Tn: CD45^+^CD3^+^CD20^–^CD4^+^CD45RA^+^CCR7^+^. CD4^+^ T effector memory: CD45^+^CD3^+^CD20^–^CD4^+^CD45RA^–^CD27^lo^CCR7^lo^. CD4^+^ Tcm: CD45^+^CD3^+^CD20^–^CD4^+^CD45RA^–^CD27^hi^CCR7^hi^. CD4^+^ Tregs: CD45^+^CD3^+^CD20^–^CD4^+^CD45RA^–^CD127^lo^CD25^+^. CD8^+^ Tn: CD45^+^CD3^+^CD20^–^CD8^+^CD45RA^+^CCR7^+^. CD8^+^ T effector memory: CD45^+^CD3^+^CD20^–^CD8^+^CD45RA^–^CD27^lo^CCR7^lo^. CD8^+^ Tcm: CD45^+^CD3^+^CD20^–^CD8^+^CD45RA^–^CD27^hi^CCR7^hi^. CD8^+^ Temra: CD45^+^CD3^+^CD20^–^CD8^+^CD45RA^+^CD27^lo^CCR7^lo^. Naive B cells: CD45^+^CD3^–^CD20^+^CD27^–^IgM^hi^IgG^–^. Memory B cells: CD45^+^CD3^–^CD20^+^CD27^+^IgM^lo^.

### Flow cytometry/FACS.

Flow data were acquired on ACSCelesta, FACSAria Fusion (BD Biosciences), or Attune Acoustic Focusing Cytometer (Invitrogen) instruments. FACS was carried out using a FACSAria Fusion. A minimum of 150 cells were used to determine heteroplasmy in antigen-specific B cells, while more than 2,000 cells were sorted for all other human/mouse subsets of interest. Flow/FACS data were analyzed using FlowJo v.10 software (TreeStar Inc.).

### In vitro stimulation of mouse T and B cells.

Naive CD4^+^ or CD8^+^ T cells and B cells were enriched from splenocytes isolated from 2-month-old mice, as described above. Isolated Tn cells were stained with Cell Trace Violet and 250,000 cells per well were seeded on 96-well U-bottom plates with/without a 1:1 (cell/bead) ratio of Mouse T-Activator CD3/CD28 beads (Invitrogen) in complete RPMI-1640 (with 10% FBS, 2.05 mM L-glutamine [Sigma-Aldrich], 1% penicillin/streptomycin [Sigma-Aldrich], 100 U/mL IL-2 [Peprotech], 55 μM 2-ME [Gibco]). For some experiments, naive CD8^+^ and CD4^+^ T cells isolated by pan-naive T cell kit (StemCell Technologies) were also stimulated by plate-bound anti-CD3/anti-CD28, in complete RPMI-1640 media with no glucose while supplemented with 10 mM galactose. For naive CD8^+^ and CD4^+^ T cell in vitro stimulation in hypoxic conditions, cells were activated and cultured in 1% O_2_ balanced with N_2_ in a tri-gas incubator. Polyclonal T cell stimulation of whole spleen suspensions was done using plate-bound anti-CD3 (5 μg/mL; clone 145-2C11, BD Biosciences) and anti-CD28 (2 μg/mL; clone 37.51, BioLegend) in 24-well plates at a density of 2 × 10^6^ cells/well. In restimulation experiments, PMA/ionomycin was added for 1 hour before protein transport was inhibited for 4 hours (1× Cell stimulation/Inhibitor Cocktail, eBioScience). Samples were then washed and stained for intracellular antigens before acquisition.

For clonal expansion experiments, modified from Lemaître et al. (65), purified naive CD8^+^ T cells from 2-month-old C5024T mice were activated using 1:1 ratio of anti-CD3/anti-CD28 Dynabeads (Invitrogen) in the presence of 100 U/mL rmIL-2. After 24-hour activation in the pool, CD8^+^ T cells were plated (in the presence of IL-2) by limiting dilution (0.8 cell/well) into 96-well (U-bottom) plates precoated with anti-CD3/anti-CD28. Each well was examined under a microscope and only wells containing a single cell were marked for further analysis. The 4 corner wells of each plate were seeded with 5,000 cells, serving as pooled activation wells. After 7 days of clonal expansion, marked wells were imaged for cell counting and lysed, before DNA extraction and pyrosequencing.

Purified B cells (250,000) were labeled with Cell Trace Violet before activation with 6 μg/mL anti-IgM F(ab)′_2_ (115-006-020, Jackson ImmunoResearch), anti-CD40 (HM40-3, 1 μg/mL, BD Biosciences), and IL-4 (20 ng/mL, R&D Systems) in complete RPMI-1640 culture media in 48-well plates.

### BMDM generation/differentiation.

Femurs were dissected intact and kept in ice-cold DMEM (Cytiva HyClone) with 0.2% FBS. Bone marrow cells were collected by flushing the cavity before 70 μm filtration. Cells were washed and centrifuged before being resuspended in DMEM with 20% FCS, 100 U/mL penicillin/streptomycin, 2 mM L-glutamine, 20 μM 2-ME, and 20 ng/mL recombinant mouse M-CSF in T75 flasks and incubated. Media were changed on days 4 and 7. Mature BMDMs were harvested using prewarmed EDTA at a concentration of 2 mM (30–40 minutes, 37°C) and evaluated by flow cytometry for expression of CD11b and F4/80. Confirmed mature BMDMs were then seeded in 6-well plates and after attachment, M1/M2 stimulation medium was added for another 24 hours. M1 polarization: DMEM with 10% FBS, 50 ng/mL LPS (O55:B5, Sigma-Aldrich), 20 ng/mL IFN-γ. M2 polarization: DMEM with 10% FBS, 20 ng/mL IL-4, 20 ng/mL IL-10, 20 ng/mL TGF-β. All recombinant mouse cytokines were purchased from Peprotech.

### Extracellular metabolic flux assay.

The Seahorse XFe96 Analyzer (Seahorse Bioscience) was used to measure OCR and ECAR in B-1a and B-2 cells, CD8^+^ T cells, and macrophages, as previously described (66, 67). Briefly, for nonadherent lymphocytes, cells were washed with Seahorse XF RPMI assay medium, before 200,000 cells/well were seeded (in triplicate) in 40 μL medium in XF 96-well cell culture microplates coated with poly-D-lysine (Sigma-Aldrich). The plate was centrifuged at 300*g* for 5 seconds with no brake, rotated 180°, and centrifuged again for 5 seconds at 300*g*. After centrifugation, 140 μL of assay medium was added per well and the plate was left to stabilize in a 37°C, non-CO_2_ incubator for 40 minutes. For BMDMs, 30,000 cells were seeded and cultured for 24 hours with IFN-γ, LPS, or IL-4 to polarize them into M1 or M2 subsets. During Seahorse assays, wells were sequentially injected with compounds to achieve final concentrations of 1,264 μM oligomycin, 2 μM FCCP, 0.5 μM rotenone, and 0.5 μM antimycin A (all from Sigma-Aldrich). OCR and ECAR were measured for each well 3 times, every 3 minutes, before and after each injection. OCR was normalized to protein concentration using a BCA Protein Assay kit (Thermo Fisher Scientific).

### Northern blot.

To study steady-state levels of mt-tRNAs, 1.2 μg of total cell RNA from BMDMs was resolved in a 10% Novex TBE-Urea gel in 1× TBE buffer (Invitrogen). The RNA was then transferred onto a Hybond-N+ Nylon membrane (GE Healthcare) at 30 V for 1 hour in 0.5× TBE. RNA was cross-linked to the membrane with 1.5 J/cm^2^ of ultraviolet radiation. Oligonucleotides for detecting mt-tRNA^ALA^ (5′-GACTTCATCCTACATCTATTG-3′) and mt-tRNA^CYS^ (5′-TCTCTACACCTTCGAATTTG-3′) were radiolabeled with [γ-^32^P]-ATP at the 5′ end using T4 polynucleotide kinase (New England Biolabs). To detect the 5.8S cytosolic rRNA (loading control), 5.8S rRNA was PCR amplified and gel purified (Qiagen). The dsDNA obtained was used as a template to prepare the DNA probe with radioactive [α-^32^P]-dCTP using the Prime-It II random primer labeling kit (Agilent). Probe hybridization temperatures for mt-tRNAs and 5.8S rRNA were 42°C and 65°C, respectively. Hybridized membranes were exposed on a phosphorimager screen and band intensity was quantified using ImageJ (NIH).

### Western blotting for OXPHOS complexes.

BMDMs were lysed in RIPA buffer containing protease and phosphatase inhibitors (cOmplete and PhosSTOP, Roche). Protein concentrations were determined by BCA (Pierce, Thermo Fisher Scientific). Equal amounts of protein (30 μg) were resolved in 4%–12% SDS-PAGE Bis-Tris gels (Thermo Fisher Scientific) and transferred to PVDF membranes (0.45 μm, Immobilon-P, Sigma-Aldrich) using the iBlot2 system (Invitrogen). Nonspecific binding was blocked with PBST containing 5% nonfat milk, followed by overnight incubation at 4°C with Total OXPHOS Rodent WB Antibody Cocktail (Abcam), followed by HRP-coupled goat anti–mouse IgG for 1 hour at room temperature. β-Actin (Cell Signaling Technology) was used as loading control. Proteins were detected using Clarity Western ECL Substrate (Bio-Rad, 170-5061). Protein bands were quantified with ImageJ.

### Quantification of mtDNA point mutation heteroplasmy levels.

Heteroplasmy levels of C5024T (mouse) or A3243G (human) mtDNA point mutations were determined by pyrosequencing, as previously described (25). Briefly, for the C5024T mutation, a 178–base pair mtDNA fragment spanning the C5024T point mutation was PCR amplified using a biotinylated forward primer (5′-TTCCACCCTAGCTATCATAAGC-3′) and a nonbiotinylated reverse primer (5′-GTAGGTTTAATTCCTGCCAATCT-3′). For the human mutation the following primers were used: forward, nonbiotinylated 5′-CCTCCCTGTACGAAAGGACA-3′; reverse, biotinylated 5′-TGGCCATGGGTATGTTGTTA-3′. After adding Streptavidin Sepharose High-Performance beads (GE Healthcare), PCR products were purified and denatured using a PyroMark Q24 vacuum workstation (Qiagen). Sequencing was carried out with PyroMark Gold Q24 reagents and sequencer (Qiagen) according to the manufacturers’ recommendations and using the sequencing primers 5′-TGTAGGATGAAGTCTTACA-3′ (for mouse) and 5′-GGTTTGTTAAGATGGCAG-3′ (for human).

### SARS-CoV-2 S vaccination.

Ten WT and 10 C5024T male animals of 2 months of age were included; 1 C5024T animal had to be sacrificed before the end of the experiment (week 7) due to a wound. Immunogens were prepared immediately before administration. Animals were injected subcutaneously over the inguinal lymph nodes with 100 μL of 2 μg S trimers in PBS plus AddaVax (Invivogen) on day 0 (prime dose) and after 5 weeks (boost dose). Two WT and 2 C5024T mice were injected with sterile PBS for use as unvaccinated controls. Tail vein blood was collected 5 weeks after the prime dose (preboost) and 5 weeks after the boost dose (at week 10, endpoint). After blood clotting, sera were separated by centrifugation and stored at –80°C until use. Serum samples were heat inactivated for neutralization experiments.

### Anti–SARS-CoV-2 ELISA.

Mice sera collected at different time points were tested for S- and RBD-specific IgG by ELISA, as previously described (68). Briefly, 96-well ELISA plates (Nunc MaxiSorp, Thermo Fisher Scientific) were coated with freshly prepared SARS-CoV-2 S trimers or RBD (100 μL of 1 ng/μL) in PBS for 15 hours at 4°C. Plates were washed 6 times with PBS/0.05% Tween 20 and blocked using PBS/5% nonfat milk (blocking buffer, Sigma-Aldrich). Mouse serum samples were thawed at room temperature, diluted, vortexed, and incubated in blocking buffer for 1 hour (4°C) before plating to block nonspecific binding. Serum samples were incubated for 15 hours at 4°C to allow low-affinity binding interactions, before washing as before. HRP-conjugated goat anti–mouse IgG (Southern Biotech, clone 1036-05) was diluted 1:2,000 in blocking buffer and incubated with samples for 1 hour at 4°C. Plates were washed a final time before development with TMB Stabilized Chromogen kept at 4°C (Invitrogen). The reaction was stopped using 1 M sulphuric acid and optical density (OD) values were measured at 450 nm using an Asys Expert 96 ELISA reader (Biochrom Ltd.). Secondary goat anti–mouse IgG (Southern Biotech, clone 1036-05) was used at 1:2,000 dilution. All assays were developed for their fixed time, and negative control samples (unvaccinated mice sera) were run alongside test samples. All samples were run at 1:100 serum dilution, unless otherwise indicated. Human MELAS Patient B anti-S and -RBD IgG reactivity was analyzed by ELISA as previously described (69). Total IgG levels were measured as previously described (70), and as above. In vitro virus neutralization assays were carried out as previously described (50). The neutralization assay limit of detection was at 1:45 serum dilution and ID_50_ titers were calculated by fitting a 4-parameter logistic curve.

### Transcriptomic profiling.

Metabolic transcriptomic signatures of CD8^+^ T cells were profiled in samples from 4 WT and 4 C5024T mice. Mice were age (2 months old) and sex matched. Briefly, for each mouse, freshly purified (negative selection) splenic naive CD8^+^ T cells were seeded at a density of 200,000 cells/well and activated using a 1:1 (bead/cell) ratio with Dynabeads Mouse T-Activator CD3/CD28 for 6 and 96 hours at 37°C (5% CO_2_) in the presence of IL-2. Ex vivo–purified naive CD8^+^ T cells (200,000) were collected for baseline (0 hour) measurement. Total RNA was extracted using TRIzol LS reagent (Invitrogen) and RNA concentrations determined using a NanoDrop ND-1000 UV-Vis Spectrophotometer (Thermo Fisher Scientific). The 6-hour time point for 1 C5024T sample failed QC (due to low RNA binding) and was excluded from the analyses.

A 768-gene panel covering 34 annotated pathways involved in 5 important metabolism themes (mouse nCounter Metabolic Pathways Panel, nanoString Technologies) was used to explore metabolic transcriptome signatures of CD8^+^ T cells in vitro after TCR activation. Total RNA (200 ng) was incubated at 65°C for 24 hours to hybridize with reporter and capture probes. The RNA complexes were subsequently immobilized and counted on an nCounter analyzer (nanoString Technologies) according to the manufacturer’s instructions. Raw data were normalized and analyzed in nSolver 4.0 (nanoString Technologies). Gene module analysis, extracting pathway-level information from a group of genes using the first principal component of their expression data (71), was used to compare genotypes.

### qPCR.

For qPCR of mouse BMDMs*,* RNA was obtained from TRIzol samples are previously described. RNA (500 ng) was reverse transcribed using the High-Capacity cDNA Reverse Transcription Kit (Thermo Fisher Scientific) in a 20 μL reaction. Twenty microliters of cDNA was diluted to 100 μL and 1 μL cDNA was used for the real-time quantitative PCR with Platinum SYBR Green qPCR SuperMix-UDG (Thermo Fisher Scientific) and QuantStudio 6 Flex PCR machine (Thermo Fisher Scientific). The following primers (5′–3′) were used: *Arg1* F: CTCCAAGCCAAAGTCCTTAGAG; R: AGGAGCTGTCATTAGGGACATC. *Il1b* F: GCAACTGTTCCTGAACTCAACT; R: ATCTTTTGGGGTCCGTCAACT. *Mhc-II (IA)* F: GTGGTGCTGATGGTGCTG; R CCATGAACTGGTACACGAAATG. *Gapdh* F: TGAAGCAGGCATCTGAGGG; R: CGAAGGTGGAAGAGTGGGAG. *Actb* F: GGCTGTATTCCCCTCCATCG; R: CCAGTTGGTAACAATGCCATGT.

### Serum cytokine analysis.

Serum IFN-γ, IL-1β, IL-2, IL-4, IL-5, IL-6, IL-10, CXCL1, IL-12p70, and TNF were measured in parallel according to the manufacturer’s recommendation using the MSD electrochemiluminescence platform (MesoScale Discovery).

### Statistics.

Statistical analyses were carried out in Prism 9 (GraphPad) and results are represented as mean ± SEM. As indicated in the figure legends, Wilcoxon’s matched-pairs signed-rank tests, unpaired *t* tests, and Mann-Whitney tests were used to analyze the data. Welch’s correction was applied when groups had unequal SD. Bonferroni’s correction was applied when performing multiple comparisons, as well as for nanoString data. A *P* value of less than 0.05 was considered significant.

### Study approval.

Ethical approval for the use of peripheral blood samples and clinical information from human MELAS patients was granted by Swedish Ethical Review Authority (registration number 2018/1498-31/3). All participants provided written informed consent. MELAS patient age boundaries are reported to preserve anonymity.

Ethical approval for investigation of C5024T mice, including vaccination challenge, was granted by the Swedish Board of Agriculture (permit numbers 10513-2020 and 20513-2020). All human and animal studies were carried out in accordance with the guidelines and policies of Karolinska Institutet and EU legislation.

### Data availability.

Please contact the authors about materials, available upon request. Transcriptomic data are deposited in the NCBI Gene Expression Omnibus (GEO GSE182887). All raw data are presented in the Supplemental Data Values.

## Author contributions

JZ, GBKH, XCD, and JR designed the study and wrote the manuscript with input from coauthors. JZ, XCD, and JR carried out wet-lab experiments and analyzed the data in consultation with coauthors. A Wedell, A Wredenberg, and ME recruited MELAS patients. CK assisted with animal experiments, along with ST, JH, QS, KZ, and YL. M Àdori, M Aoun, and LB assisted with flow cytometry. RF assisted with pyrosequencing. LH, BM, and GM provided SARS-CoV-2 antigens and reagents. DJS carried out SARS-CoV-2 neutralization assays. SG carried out Northern blotting. RAH, RH, and MP provided critical reagents and advised on different aspects of the project.

## Supplementary Material

Supplemental data

Supplemental table 1

Supporting data values

## Figures and Tables

**Table 1:**
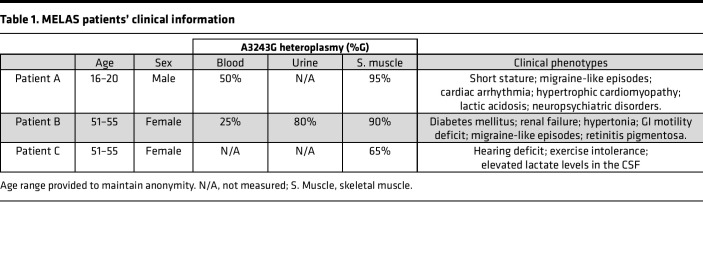
MELAS patients’ clinical information

## Version 1 09/08/2023

Electronic publication
