# Supplementary material for: Antigen receptor stimulation induces purifying selection against pathogenic mitochondrial tRNA mutations
Source: JCI Insight. 2023 Sep 8;8(17):e167656. doi: 10.1172/jci.insight.167656 (PMC10544217; doi:10.1172/jci.insight.167656)
Supplement: Supplemental data [file jciinsight-8-167656-s126.pdf]

Supplementary Figures (1-11)

Antigen receptor stimulation induces purifying selection against pathogenic mitochondrial tRNA mutations

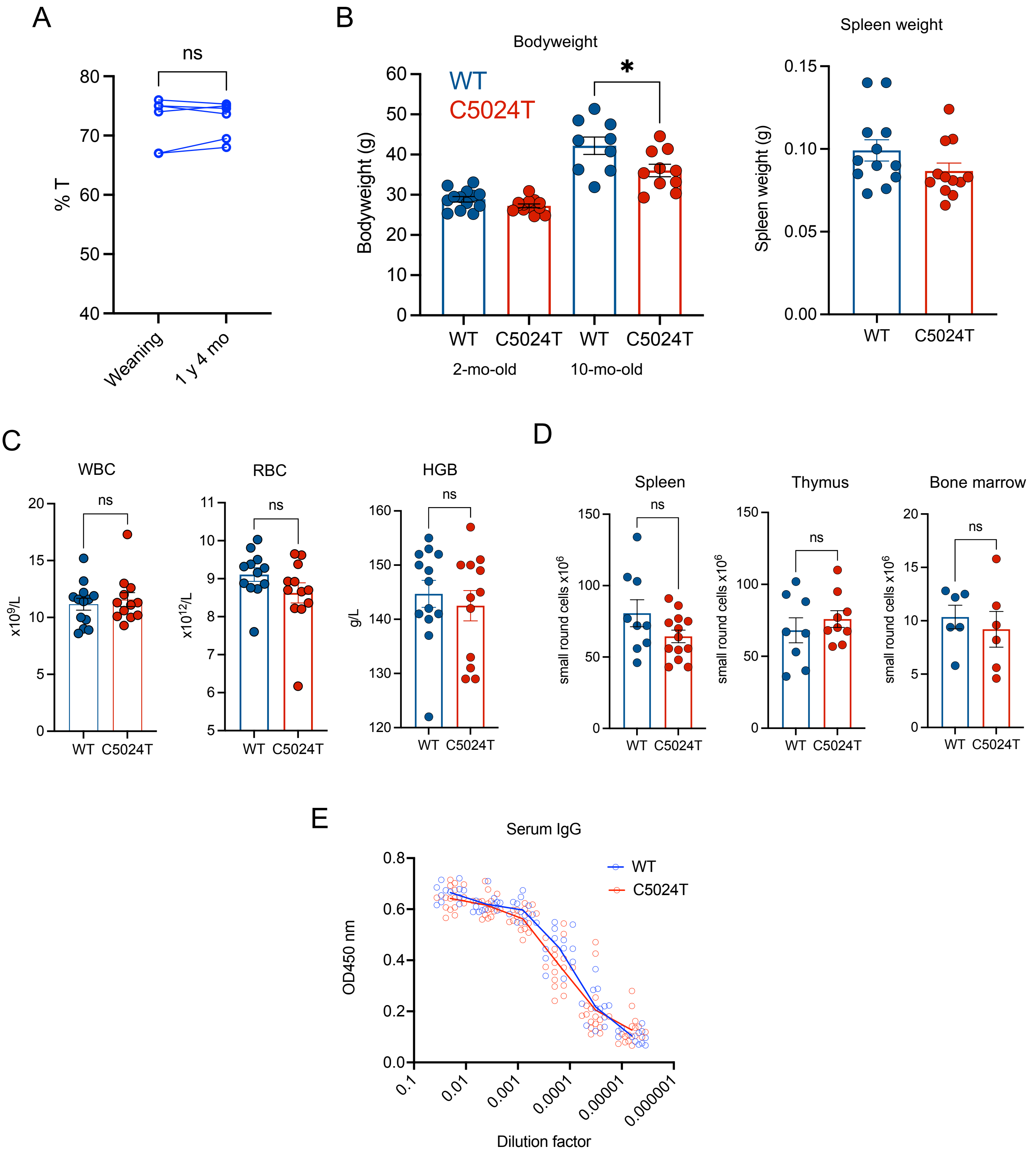

Figure S1 – Baseline C5024T phenotypes

A. C5024T heteroplasmy in ear biopsies collected at weaning and 1-year-4-month-of-age in the same 5 C5024T mice.

B. Bodyweight and spleen weight at termination are shown for C5024T and WT controls. Spleen weight was recorded only in 2-month-old animals.

C. Total white blood cell (WBC), red blood cell (RBC) and hemoglobin (HGB) concentrations in blood in C5024T and WT mice 2-months-of-age.

D. The cellularity of single cell suspensions from spleen, thymus and femur flushes are shown for C5024T and WT mice 2-months-of-age.

E. Serum IgG titers (determined by ELISA) were not significantly different between C5024T (n = 16) and *wt* mice (n = 12) at 2-months-old-age. A multiple unpaired t-test with Welch correction was used to analyze these data in (E).

Figure S1

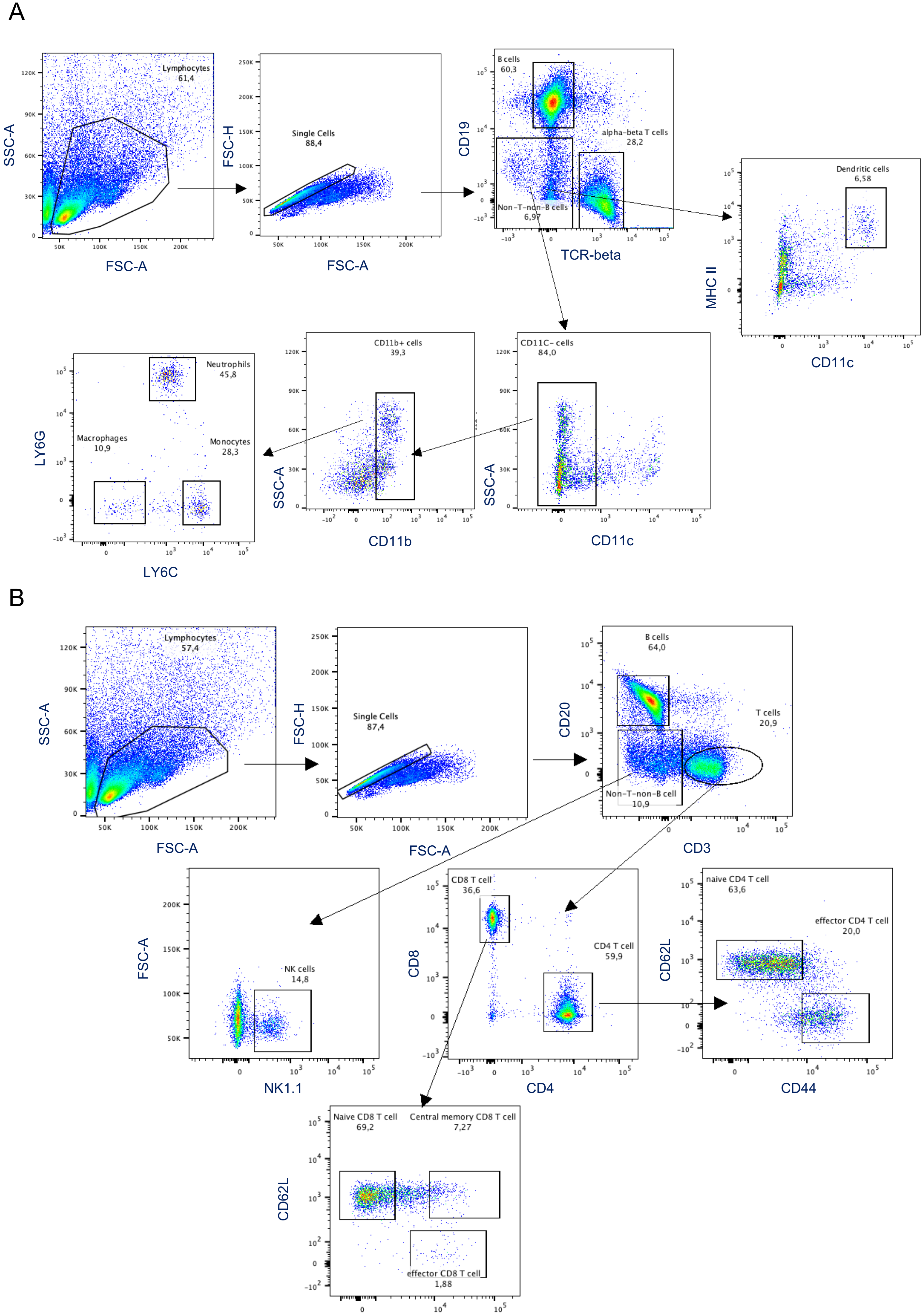
A
Lymphocytes 61,4
SSC-A
FSC-A
Single Cells 88,4
FSC-H
FSC-A
B cells 60,3
alpha-beta T cells 28,2
Non-T-non-B cells 6,97
CD19
TCR-beta
Dendritic cells 6,58
MHC II
CD11c
CD11C- cells 84,0
SSC-A
CD11c
CD11b+ cells 39,3
SSC-A
CD11b
Neutrophils 45,8
Macrophages 10,9
Monocytes 28,3
LY6G
LY6C
B
Lymphocytes 57,4
SSC-A
FSC-A
Single Cells 87,4
FSC-H
FSC-A
B cells 64,0
T cells 20,9
Non-T-non-B cell 10,9
CD20
CD3
NK cells 14,8
FSC-A
NK1.1
CD8 T cell 36,6
CD4 T cell 59,9
CD8
CD4
naive CD4 T cell 63,6
effector CD4 T cell 20,0
CD62L
CD44
Naive CD8 T cell 69,2
Central memory CD8 T cell 7,27
effector CD8 T cell 1,88
CD62L
CD44

Figure S2

Figure S2 – Mouse FACS gating strategy for myeloid and T cell subsets

Down-sampled event gates are shown.

A. FACS gating strategy for myeloid subsets of interest isolated from C5024T mice.

B. FACS gating strategy for T cell subsets of interest isolated from C5024T mice. *B cells are shown in Fig. 3, as well as antigen-specific gates. Markers used to define each subset are listed in the Materials and Methods.*

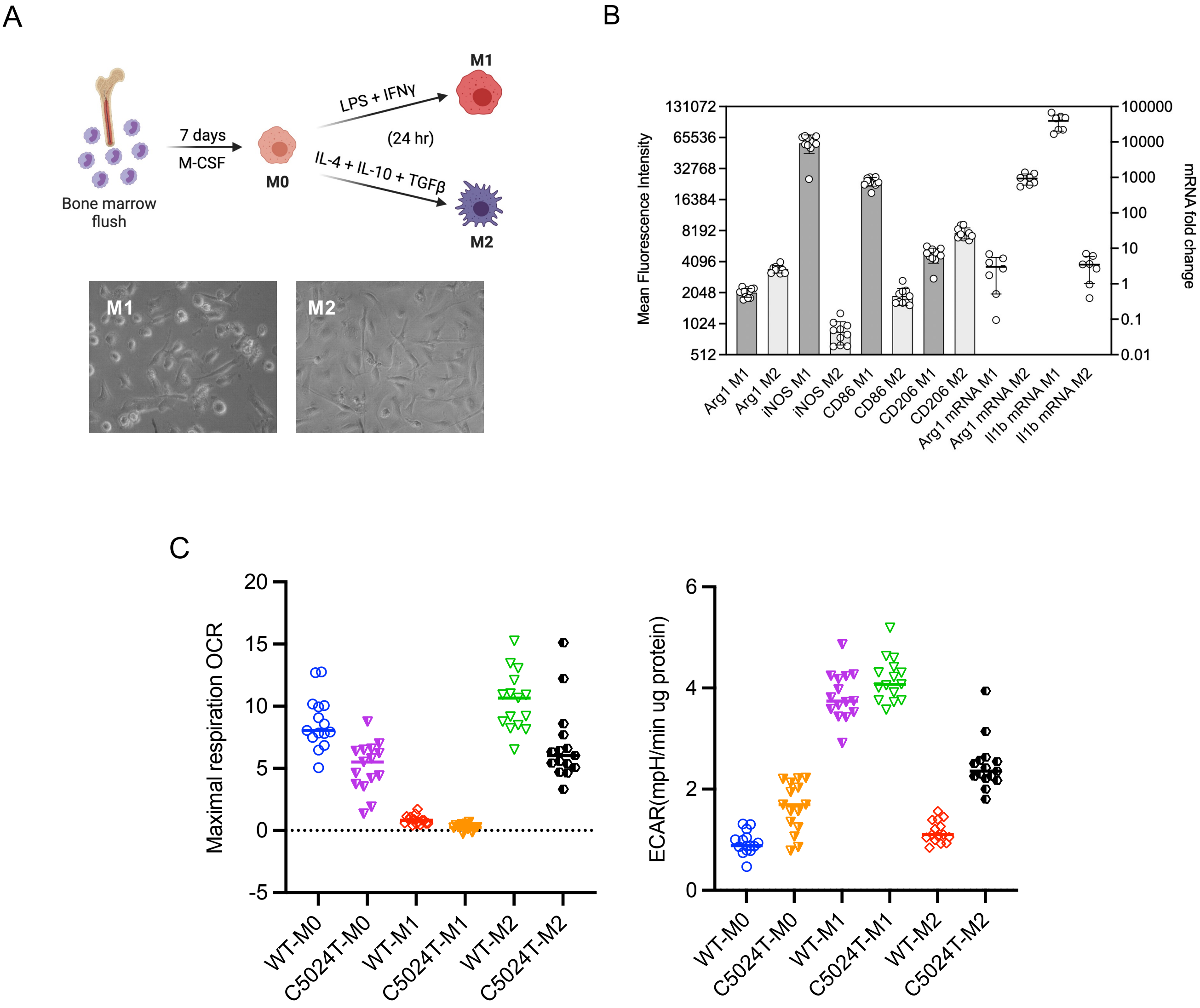

Figure S3 – *In vitro* bone marrow-derived macrophage generation and phenotyping

A. Schematic for the generation of M0, M1 and M2 BMDM from mouse femur. Cell morphological differences are shown in the inset images for polarized cells.

B. Confirmatory protein and mRNA (qPCR) phenotyping of M1 and M2 polarization conditions. M1 cells increased iNOS, CD86 and IL-1-beta expression, while M2 cells increased Arg1 and CD206, in agreement with morphological differences shown in A (minimum *n*=4).

C. Maximal respiration (OCR) in M0, M1 and M2 cells, 24 h after polarization, separated by genotype, and Basal ECAR in M0, M1 and M2 cells, 24 h after polarization, separated by genotype.

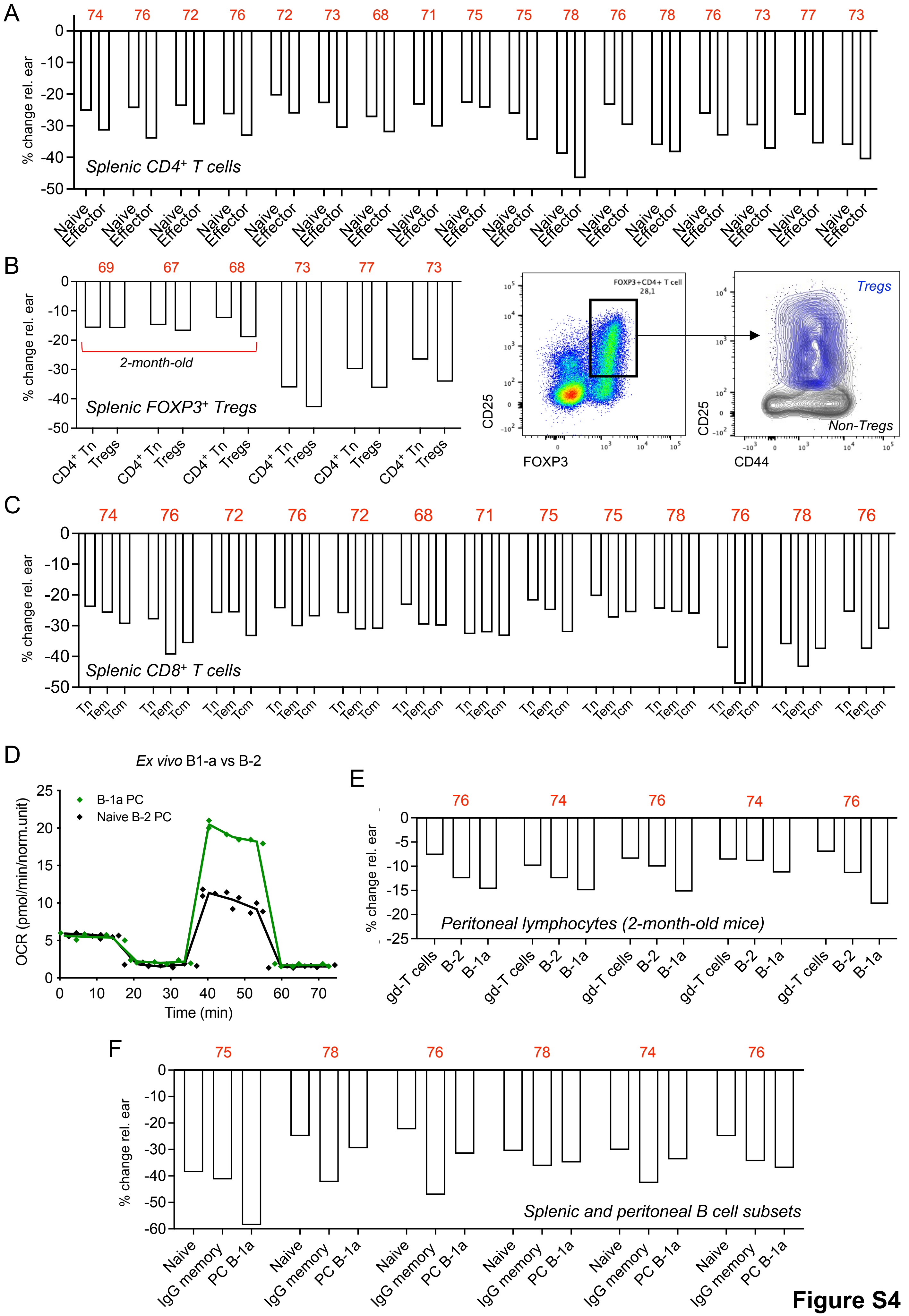
A
% change rel. ear
Splenic CD4⁺ T cells
74 76 72 76 72 73 68 71 75 75 78 76 78 76 73 77 73
Naive
Effector
B
69 67 68 73 77 73
2-month-old
Splenic FOXP3⁺ Tregs
CD4⁺ Tn
Tregs
FOXP3+CD4+ T cell
28,1
CD25
FOXP3
Tregs
Non-Tregs
CD44
C
74 76 72 76 72 68 71 75 75 78 76 78 76
Splenic CD8⁺ T cells
Tn
Tem
Tcm
D
Ex vivo B1-a vs B-2
B-1a PC
Naive B-2 PC
OCR (pmol/min/norm.unit)
Time (min)
E
76 74 76 74 76
Peritoneal lymphocytes (2-month-old mice)
gd-T cells
B-2
B-1a
F
75 78 76 78 74 76
Splenic and peritoneal B cell subsets
Naive
IgG memory
PC B-1a

Figure S4

Figure S4 – Mouse T and B cell subsets pyrosequencing results

Ear punch biopsy heteroplasmy (percentage) at weaning is shown in red for each animal used.

A. Heteroplasmy (%T vs. ear) is shown in sorted splenic $CD4^+$ naïve and effector memory cells from individual animals (*n*=17).

B. Heteroplasmy (%T vs. ear) is shown in sorted splenic $CD4^+$ naïve and Tregs from individual animals (*n*=3 adult, 3 aged). Inset plot showing CD44 expression on the sorted FOXP3+CD25+ subset of Tregs. *Data representative of all animals from which Tregs were sorted.*

C. Heteroplasmy (%T vs. ear) is shown in sorted splenic $CD8^+$ naïve, central memory and effector memory cells from individual animals (*n*=13).

D. SeaHorse Mito Stress test of ex vivo FACS-isolated B-1a and naïve B-2 cells from the peritoneum and total splenocytes.

E. Heteroplasmy (%T vs. ear) is shown in sorted peritoneal B-1a, naïve B-2 and gamma-delta T cells (*n*=5) from adult animals.

F. Heteroplasmy (%T vs. ear) is shown in sorted splenic naïve B-2, IgG^+ memory B-2, and peritoneal B-1a cells from aged animals (shown for representative *n*=6).

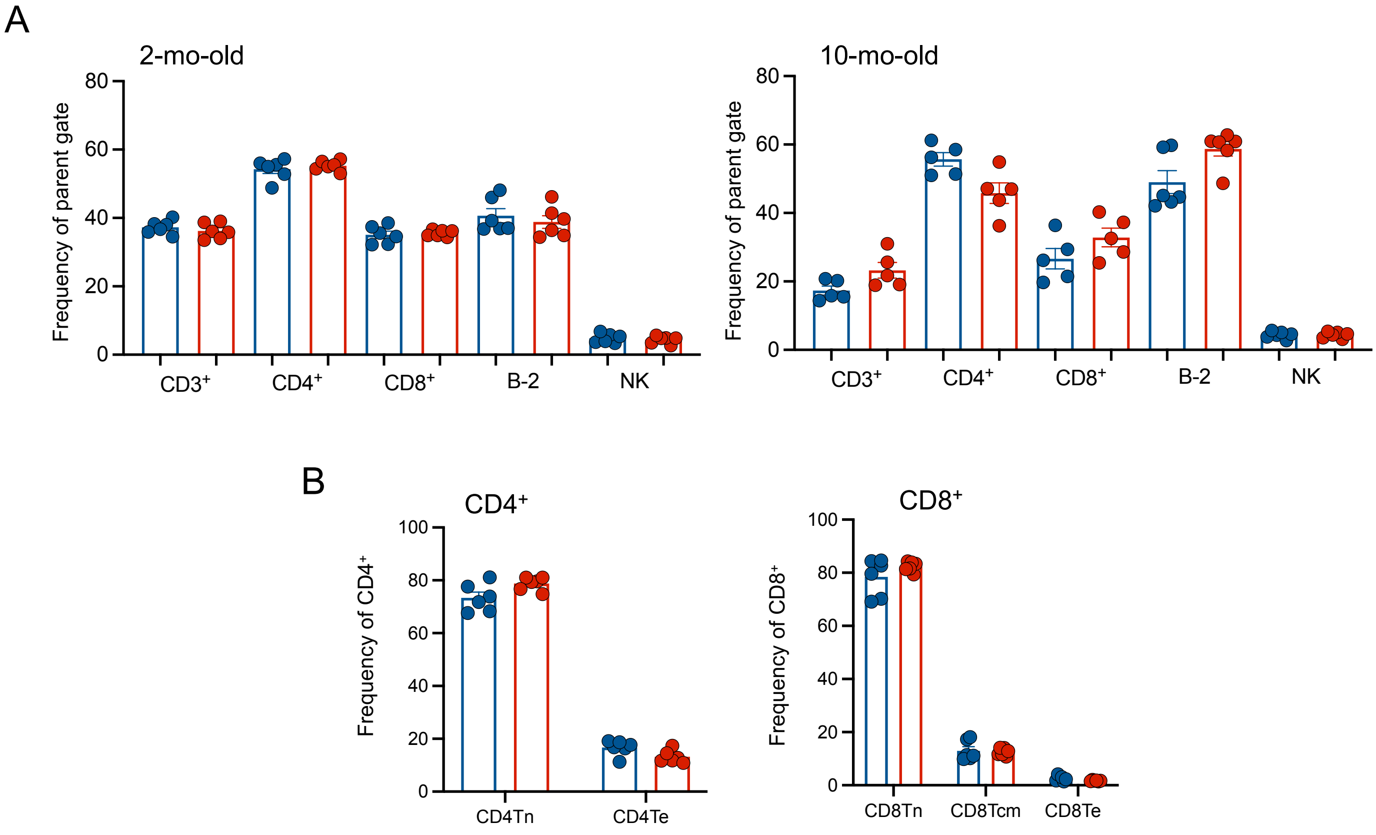

Figure S5 – Baseline immunophenotyping of C5024T mice

A. CD3+, CD4+, CD8+ T cell, B cell and NK cell frequencies in the spleens of 2- and 10-month-old C5024T and WT mice. Groups of *n* = 6 2-mo-old, *n* = 5 10-mo-old.

B. Naïve, effector, and memory T cell subsets in spleens from 10-month-old C5024T (*n*=6) and WT animals (*n*=6).

T cell data are representative of three independent experiments.

Figure S5

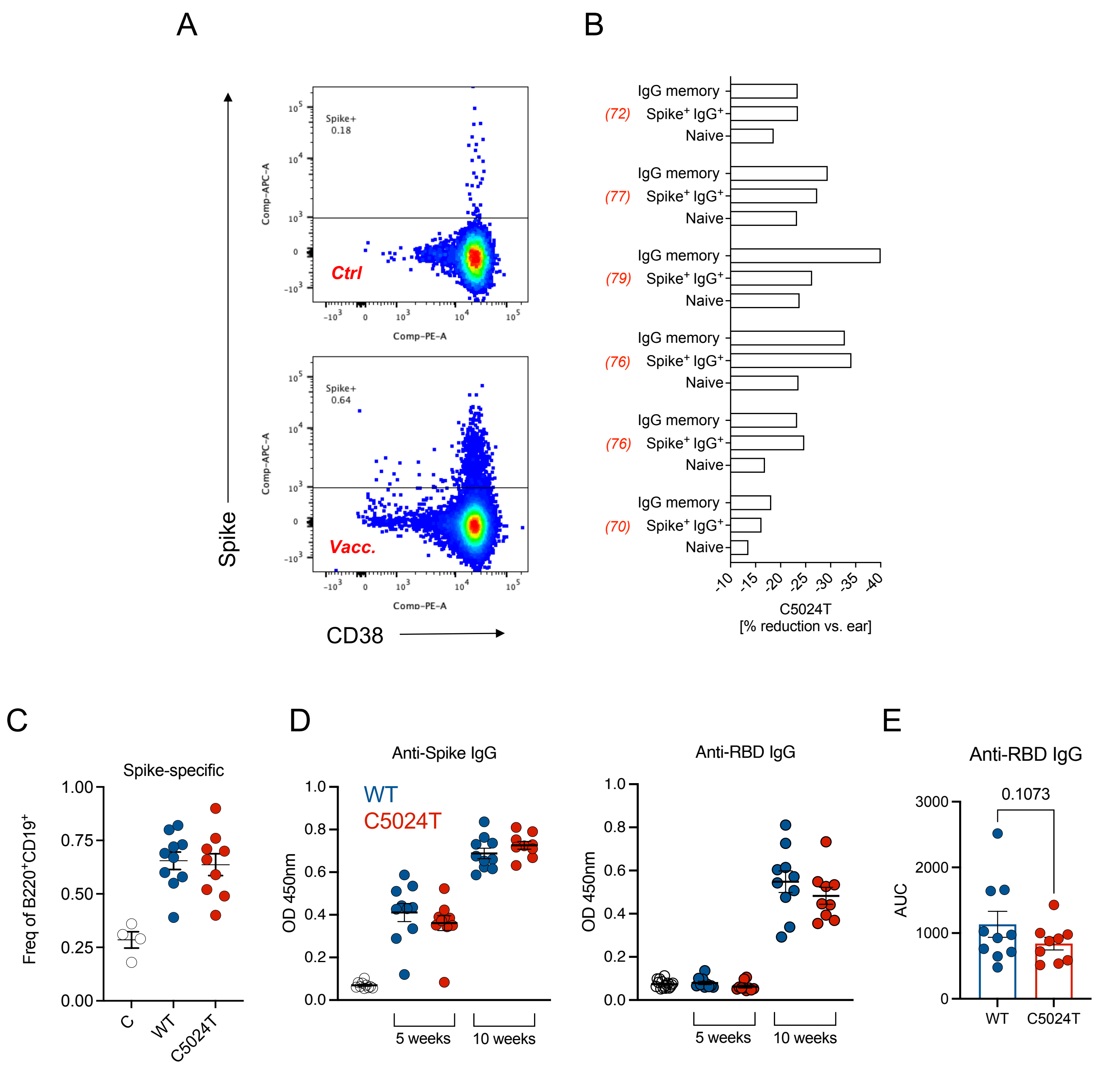

Figure S6 – Spike vaccination phenotypes

A. Representative pseudocolor dot plots from one vaccinated and one unvaccinated mice, showing the Spike signal 5 weeks post-boost.

B. Heteroplasmy (%T vs. ear) in sorted B cell subsets from vaccinated C5024T mice (*n*=6). Naïve: $CD3^{-}CD19^{+}B220^{+}IgD^{hi}IgM^{hi}IgG^{-}$; Memory (Ag-specific): $CD3^{-}CD19^{+}B220^{+}IgD^{-}IgM^{-}IgG^{+}S^{+}$; Memory (non-Ag-specific): $CD3^{-}CD19^{+}B220^{+}IgD^{-}IgM^{-}IgG^{+}Spike^{-}$. Ear heteroplasmy levels at weaning are shown in red brackets.

C. Frequency of S-specific B cells from C5024T (*n*=10) and WT (*n*=9) mice plotted as the frequency of Ag-specific from total B-2 cells.

D. Anti-S and anti-RBD IgG 5 weeks post-prime (pre-boost) and 5 weeks post-boost. Repeat analysis of unvaccinated controls (*n*=2 WT, *n*=2 C5024T) are shown as open circles.

E. Serial dilution of serum to interpret total anti-RBD IgG levels in C5024T (*n*=9) and WT mice (*n*=10) 5 weeks after the boost. 5-fold serial dilution (starting at 1:100) was used to calculate the area under the curve (AUC) for each mouse and genotypes compared.

Figure S6

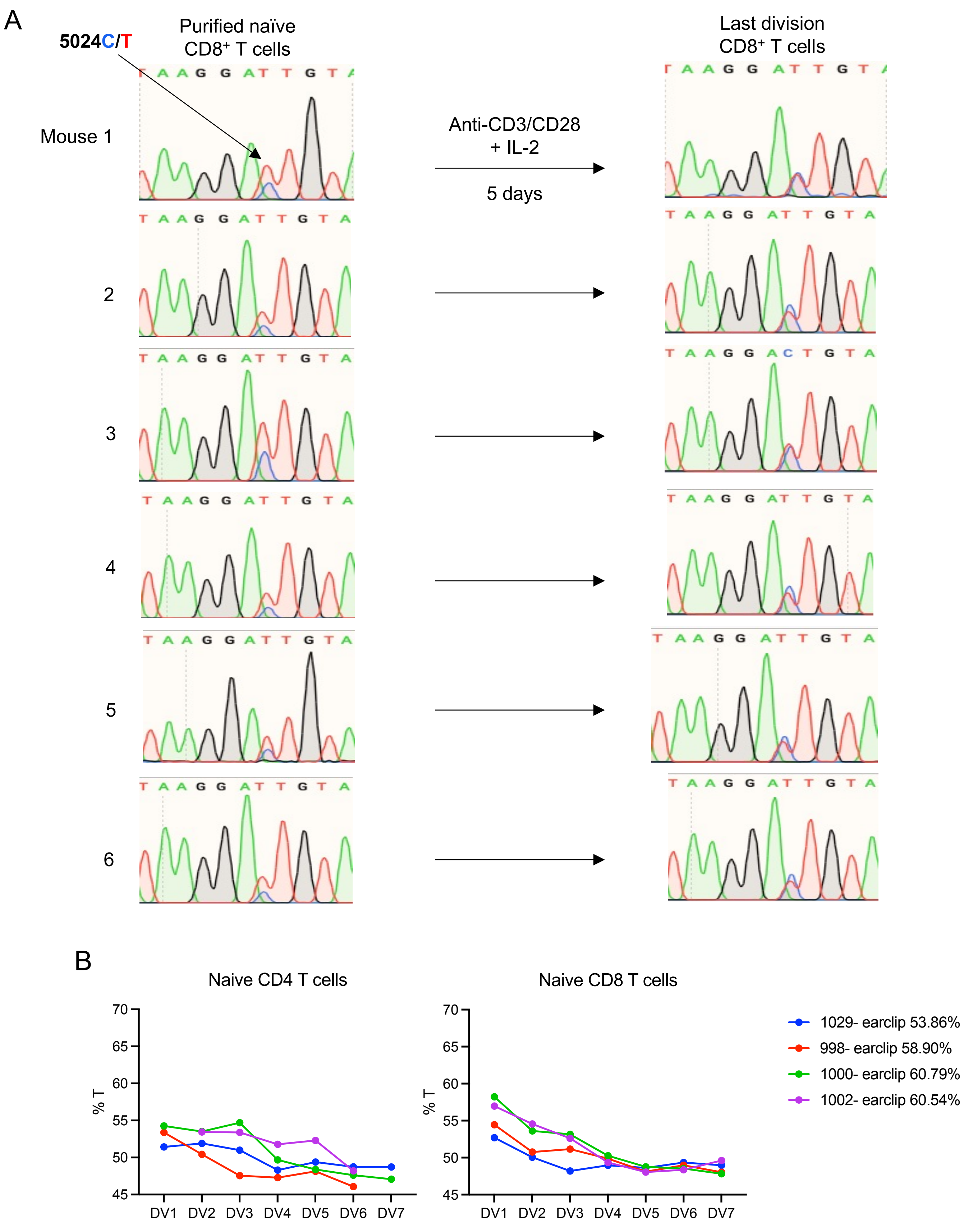
A
5024C/T
Purified naïve CD8+ T cells
Last division CD8+ T cells
Mouse 1
2
3
4
5
6
Anti-CD3/CD28 + IL-2
5 days
B
Naive CD4 T cells
Naive CD8 T cells
% T
70
65
60
55
50
45
DV1
DV2
DV3
DV4
DV5
DV6
DV7
1029- earclip 53.86%
998- earclip 58.90%
1000- earclip 60.79%
1002- earclip 60.54%

Figure S7

Figure S7 – Sanger sequencing of *ex vivo* naïve and most-divided CD8⁺ T cells after *in vitro* stimulation

A. Sanger sequencing of *ex vivo* naïve and most-divided (FACS-isolated 5 days after activation) $CD8^+$ T cells to confirm the heteroplasmy reduction observed by pyrosequencing. Results from six individual animals (> 65% T at weaning) are shown.
B. C5024T heteroplasmy in stimulated naïve CD4 and CD8 T cells isolated from animals with < 61% T in the ear at weaning. Four C5024T animals were used.

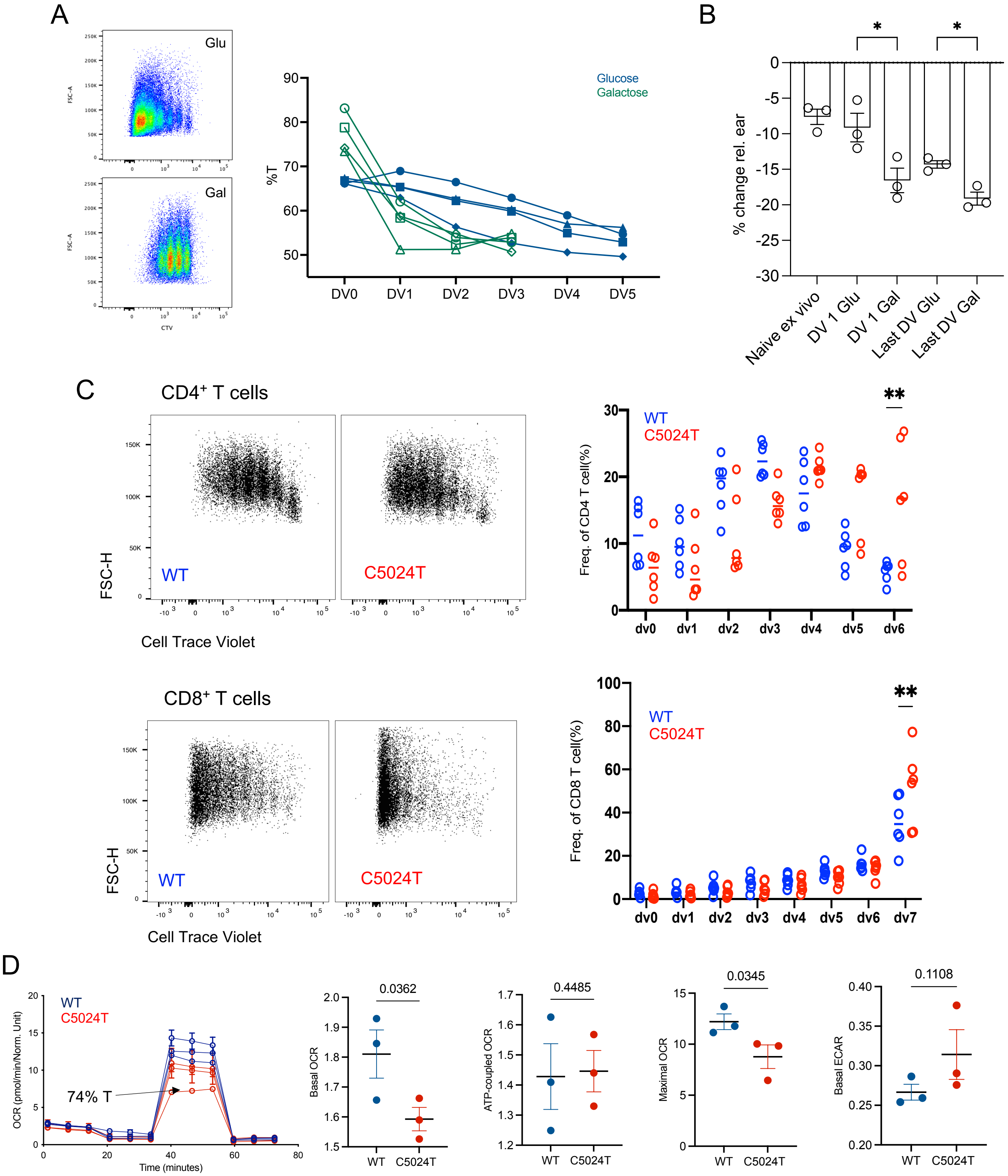
A
Glu
Gal
FSC-A
CTV
Glucose
Galactose
%T
DV0
DV1
DV2
DV3
DV4
DV5
B
% change rel. ear
Naive ex vivo
DV 1 Glu
DV 1 Gal
Last DV Glu
Last DV Gal
C
CD4+ T cells
WT
C5024T
FSC-H
Cell Trace Violet
Freq. of CD4 T cell(%)
dv0
dv1
dv2
dv3
dv4
dv5
dv6
CD8+ T cells
Freq. of CD8 T cell(%)
dv7
D
OCR (pmol/min/Norm. Unit)
74% T
Time (minutes)
Basal OCR
0.0362
ATP-coupled OCR
0.4485
Maximal OCR
0.0345
Basal ECAR
0.1108

Figure S8

Figure S8 – *In vitro*-stimulated lymphocyte phenotypes

A. Representative pseudocolor CTV plots are shown for naïve C5024T $CD8^+$ T cells TCR-stimulated under glucose/galactose conditions. %T in each division is displayed for each condition. Four C5024T mice were used for this experiment.

B. Naïve $CD4^+$ T cells were activated and cultured in glucose or galactose media and cells from the named divisions FACS-isolated for pyrsequencing. Data from *n*=3 C5024T mice.

C. Proliferation kinetics of WT (*n*=6) and C5024T (*n*=6) $CD4^+$ and $CD8^+$ naïve T cells stimulated with anti-CD3/CD28 for 5 days. *Down-sampled dot plots (from $CD4^+$ and $CD8^+$ gates) are representative of C5024T and WT group responses and were reproduced in* 3 *independent experiments.*

D. SeaHorse analysis of unstimulated *ex vivo* naïve $CD8^+$ T cells from C5024T and WT mice. % T in ear for the three C5024T mice were 68, 68 and 74%, with the highest %T highlighted. A one-tailed t-test was used to analyze the data.

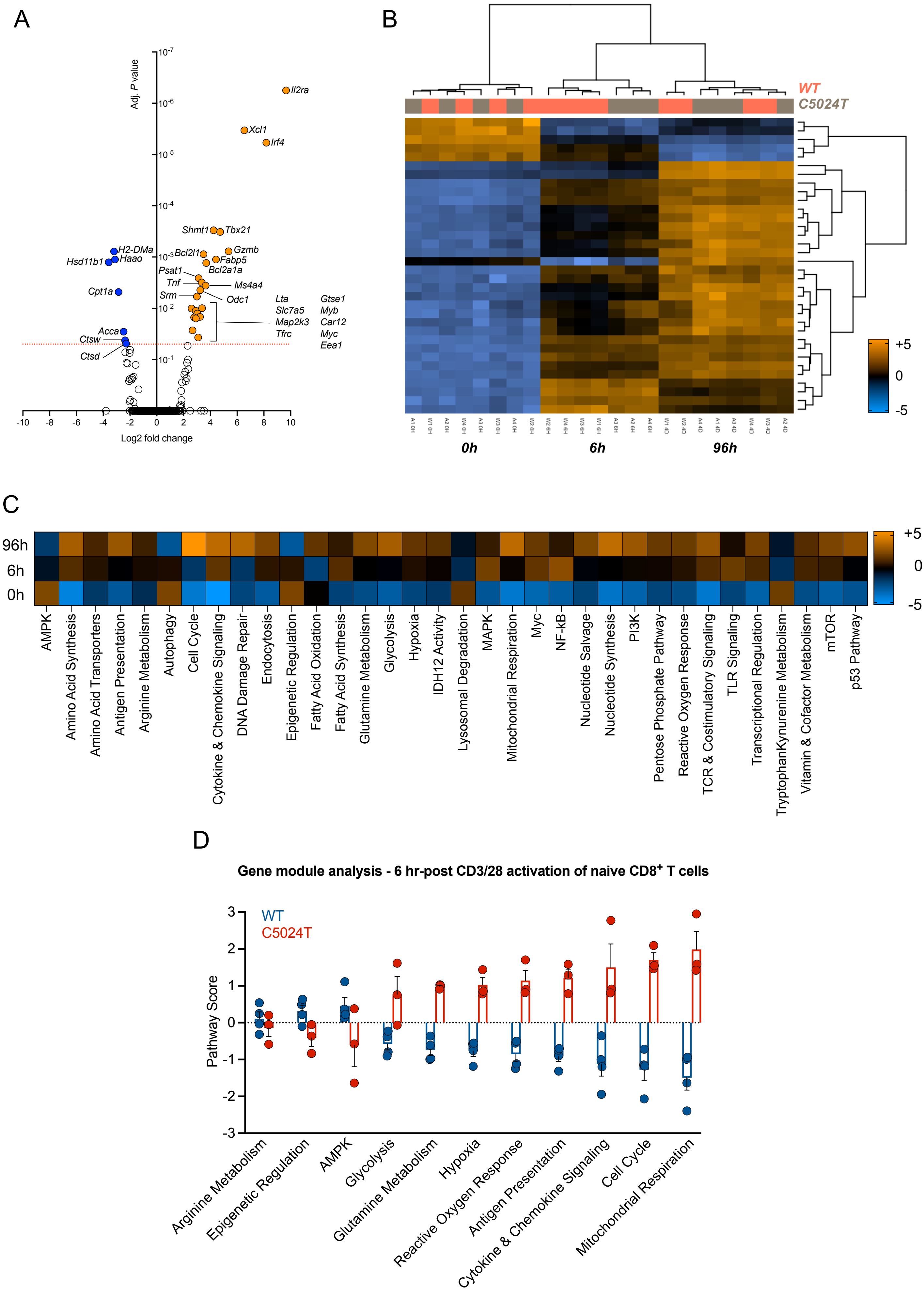
A
Adj. P value
Log2 fold change
Il2ra
Xcl1
Irf4
Shmt1
Tbx21
H2-DMa
Haao
Hsd11b1
Bcl2l1
Gzmb
Fabp5
Bcl2a1a
Psat1
Tnf
Ms4a4
Srm
Odc1
Cpt1a
Lta
Slc7a5
Map2k3
Tfrc
Gtse1
Myb
Car12
Myc
Eea1
Acca
Ctsw
Ctsd
B
WT
C5024T
0h
6h
96h
C
96h
6h
0h
AMPK
Amino Acid Synthesis
Amino Acid Transporters
Antigen Presentation
Arginine Metabolism
Autophagy
Cell Cycle
Cytokine & Chemokine Signaling
DNA Damage Repair
Endocytosis
Epigenetic Regulation
Fatty Acid Oxidation
Fatty Acid Synthesis
Glutamine Metabolism
Glycolysis
Hypoxia
IDH12 Activity
Lysosomal Degradation
MAPK
Mitochondrial Respiration
Myc
NF-kB
Nucleotide Salvage
Nucleotide Synthesis
PI3K
Pentose Phosphate Pathway
Reactive Oxygen Response
TCR & Costimulatory Signaling
TLR Signaling
Transcriptional Regulation
TryptophanKynurenine Metabolism
Vitamin & Cofactor Metabolism
mTOR
p53 Pathway
D
Gene module analysis - 6 hr-post CD3/28 activation of naive CD8+ T cells
Pathway Score
WT
C5024T
Arginine Metabolism
Epigenetic Regulation
AMPK
Glycolysis
Glutamine Metabolism
Hypoxia
Reactive Oxygen Response
Antigen Presentation
Cytokine & Chemokine Signaling
Cell Cycle
Mitochondrial Respiration

Figure S9

Figure S9 – NanoString metabolic pathway gene expression profiling

A. Volcano plot of genes differentially expressed between 0 (unstimulated) and 6 h of stimulation (in all 7 animals, regardless of genotype). Bonferroni-corrected *P*-values for differential expression are shown. One C5024T sample (from original *n*=4) failed QC for low RNA binding and was excluded from analyses.

B. Hierarchical clustering of gene module analysis over the activation time course is shown for all mice (*n*=3 C5024T, *n*=4 WT). Pathway z-scores are represented on the color scale.

C. Mean gene module pathway scores for all mice (*n*=8) are shown for each of the three timepoints.

D. Key gene modules differentially expressed between C5024T and WT animals after 6h. Differences are relative to the other genotype, i.e., C5024T shows more expression of mitochondrial respiration genes than WT cells, but both mutant and control animals express pathway genes.

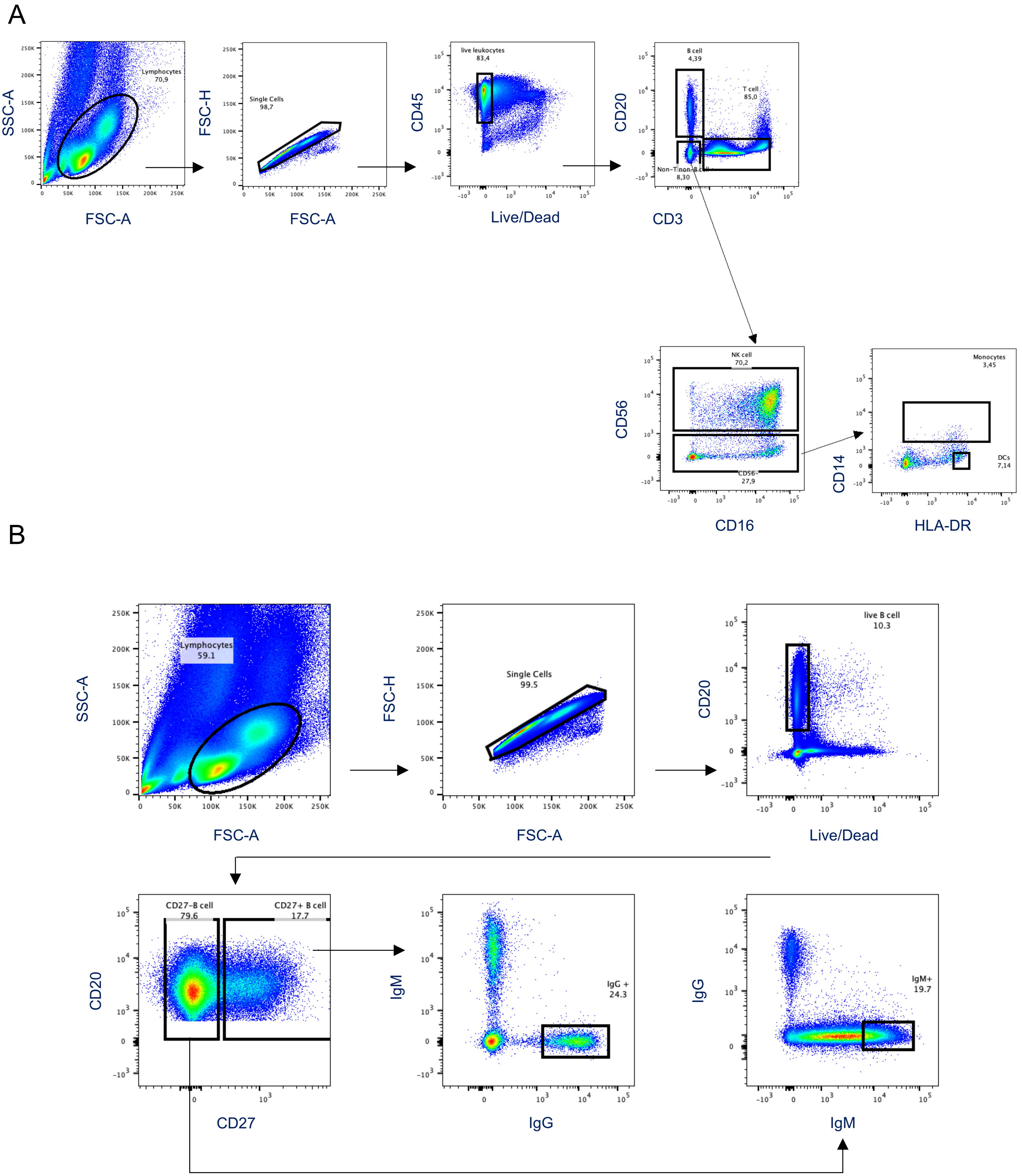

Figure S10 – Human innate and B cell FACS gating strategy

A. FACS gating strategy for isolation of human myeloid lineages from the PBMC.

B. FACS gating strategy for isolation of human B cell subsets from the PBMC.

Figure S10

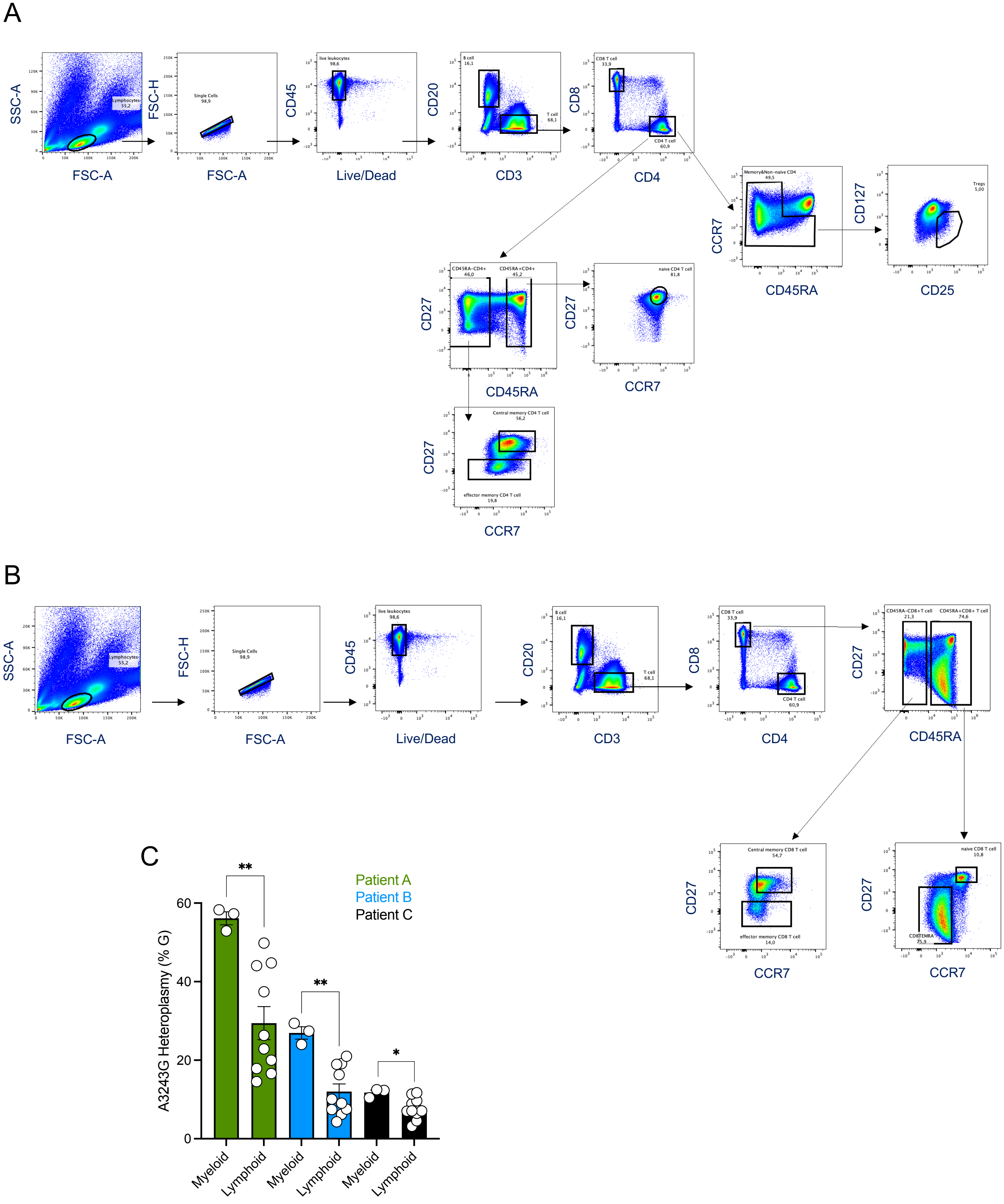

Figure S11 – Human T cell subsets FACS gating strategy and MELAS phenotypes

A. FACS gating strategy for isolation of human $CD4^{+}$ T cell subsets from PBMCs.

B. FACS gating strategy for isolation of human $CD8^{+}$ T cell subsets from PBMCs.

C. A3243G heteroplasmy in myeloid vs. lymphoid lineages shown in Fig. 6. Myeloid vs. lymphoid selection was compared within a patient. Each point represents a different cell type.

Figure S11
